# Intersection of transfer cells with phloem biology—broad evolutionary trends, function, and induction

**DOI:** 10.3389/fpls.2013.00221

**Published:** 2013-07-01

**Authors:** Felicity A. Andriunas, Hui-Ming Zhang, Xue Xia, John W. Patrick, Christina E. Offler

**Affiliations:** Department of Biological Sciences, School of Environmental and Life Sciences, The University of NewcastleCallaghan, NSW, Australia

**Keywords:** transfer cell, ingrowth wall architecture, phloem transport, inductive signals

## Abstract

Transfer cells (TCs) are ubiquitous throughout the plant kingdom. Their unique ingrowth wall labyrinths, supporting a plasma membrane enriched in transporter proteins, provides these cells with an enhanced membrane transport capacity for resources. In certain plant species, TCs have been shown to function to facilitate phloem loading and/or unloading at cellular sites of intense resource exchange between symplasmic/apoplasmic compartments. Within the phloem, the key cellular locations of TCs are leaf minor veins of collection phloem and stem nodes of transport phloem. In these locations, companion and phloem parenchyma cells *trans*-differentiate to a TC morphology consistent with facilitating loading and re-distribution of resources, respectively. At a species level, occurrence of TCs is significantly higher in transport than in collection phloem. TCs are absent from release phloem, but occur within post-sieve element unloading pathways and particularly at interfaces between generations of developing Angiosperm seeds. Experimental accessibility of seed TCs has provided opportunities to investigate their inductive signaling, regulation of ingrowth wall formation and membrane transport function. This review uses this information base to explore current knowledge of phloem transport function and inductive signaling for phloem-associated TCs. The functional role of collection phloem and seed TCs is supported by definitive evidence, but no such information is available for stem node TCs that present an almost intractable experimental challenge. There is an emerging understanding of inductive signals and signaling pathways responsible for initiating *trans*-differentiation to a TC morphology in developing seeds. However, scant information is available to comment on a potential role for inductive signals (auxin, ethylene and reactive oxygen species) that induce seed TCs, in regulating induction of phloem-associated TCs. Biotic phloem invaders have been used as a model to speculate on involvement of these signals.

## Introduction

Transfer cells (TCs) are located at “bottlenecks” for membrane transport of nutrients within plants. Their unique wall architecture and abundance of transporter proteins provides these cells with an enhanced membrane transport capacity. They occur at interfaces between plants and their environment (root-soil and, for submerged leaves, leaf-water, interfaces) and at symplasmic/apoplasmic interfaces in loading/unloading of vascular pathways and exchange between them; in developing reproductive organs; in secretory organs (nectaries, glands, and trichomes) and in association with biotic symbionts (nitrogen-fixing bacteria and mycorrhiza) and intruders (leafhoppers, nematodes, parasites) (Pate and Gunning, 1972; Offler et al., 2003). TCs have been reported for all taxa of the plant kingdom and in algae and fungi, raising the expectation that every plant has the genomic capacity to form TCs given a specific set of environmental conditions and/or developmental signals. This being the case, the potential exists for expression of this important cell type in strategic locations for nutrient flow in species where TCs are known to be absent.

Generally TCs develop from a range of differentiated cell types by a process that involves de- followed by re-differentiation referred to as *trans*-differentiation. From an evolutionary perspective, this developmental phenomenon first appears in algae (e.g., thallus cell TCs of the characean alga *Coleochaete orbicularis*—Graham and Wilcox, 1983) from which it radiated into all other plant taxa. However, in meristems within the plant axis differentiation of the host cell type and its TC identity may occur concurrently (e.g., in the hypocotyl and cotyledonary node of lettuce—Pate et al., 1970). Traditionally, TCs retain the name of the original cell type, for example, epidermal TC. The *trans*-differentiation process is either part of the plant's developmental program or occurs in response to an abiotic or biotically-created stress. The resulting TC has a characteristic intricately-invaginated ingrowth wall ensheathed by a plasma membrane enriched in nutrient transporters. The latter is abutted by a cytoplasm with high numbers of mitochondria, ribosomes and endomembrane system components (Offler et al., 2003).

**Figure 1:**
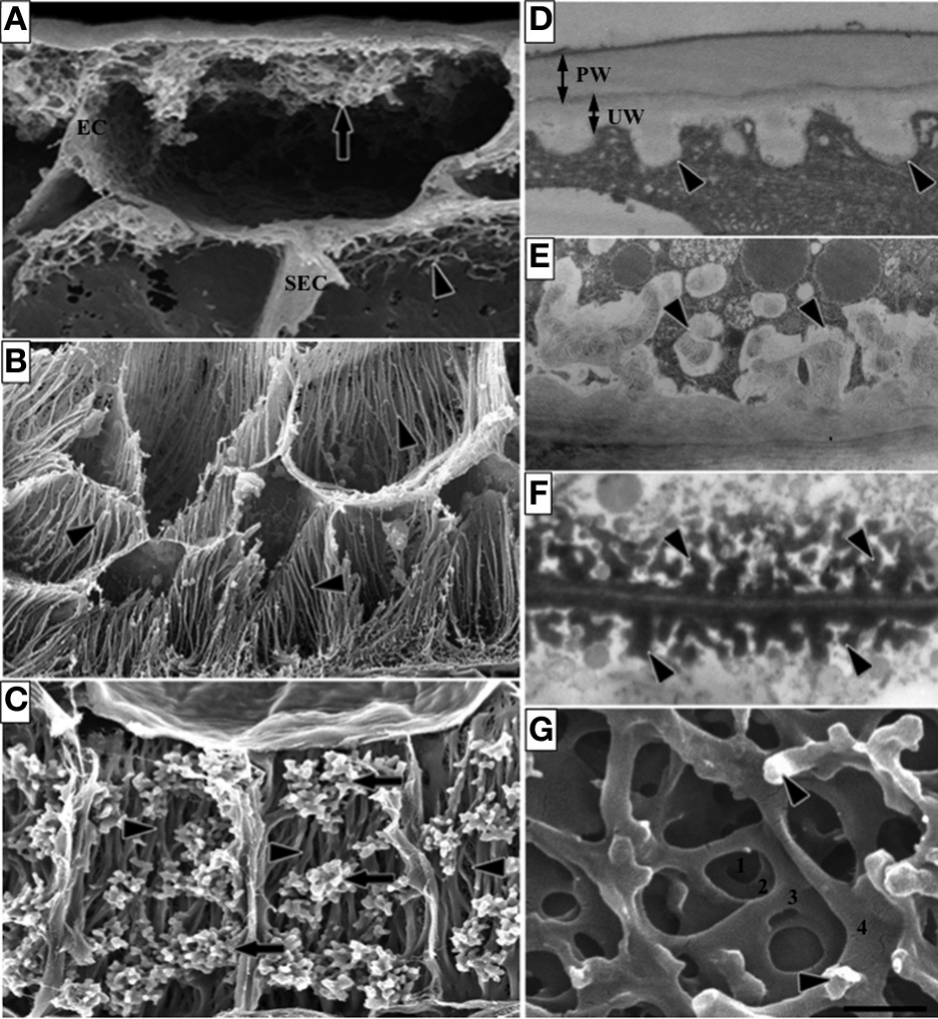
**Images of transfer cells of developing seeds during their storage phase illustrating ingrowth wall morphologies. (A–C)** Scanning **(A,B)** and field emission scanning **(C)** electron microscope images of cells following freeze-fracture, removal of their cytoplasm and fixation [for method see Talbot et al. (2002)]. **(A)** Epidermal transfer cells (ETC) of a *Vicia faba* cotyledon with an extensive reticulate ingrowth wall labyrinth (arrow) polarized to the outer periclinal wall. Ingrowth wall deposition (dart) is restricted to wall portions abutting intercellular spaces adjacent to the sub-epidermal cells (SEC) [modified after Talbot et al. (2001)]. **(B)** Basal endosperm transfer cells of *Zea mays* exhibiting flange wall ingrowth morphology [modified after Talbot et al. (2002)]. The wall ingrowth ribs (darts) extend the length of each cell and are more extensive at their outer periclinal walls. **(C)** Thin-walled parenchyma transfer cells located at the inner surface of the inner seed coat of *Gossypium hirsutum* with wall ingrowth flanges (darts) extending the length of each cell on which are deposited groups of reticulate wall ingrowths (arrows) [modified after Pugh et al. (2010)]. **(D–F)** Transmission electron microscope images of portions of transverse sections of transfer cells: **(D)** The outer periclinal wall of an adaxial epidermal cell of a *V. faba* cotyledon induced to *trans*-differentiate to a transfer cell morphology. A uniform wall (UW), distinguishable from the original primary wall (PW) by a different electron opaqueness, is deposited against the primary wall and small papillate wall ingrowths (darts) arise from it. **(E)** Small papillate ingrowths (darts) of a seed coat transfer cell of *V. faba* exhibiting reticulate architecture. **(F)** Antler-shaped reticulate wall ingrowths (darts) of a nucellar projection transfer cell of a developing *Triticum turgidum* var. *durum* seed [modified after Wang et al. (1994)]. **(G)** Field emission scanning electron microscope image of the cytoplasmic face of the reticulate ingrowth wall labyrinth of an abaxial epidermal transfer cell of a *V. faba* cotyledon following removal of the cytoplasm and dry cleaving [for method see Talbot et al. (2001), image modified after Talbot et al. (2001)]. Note the multi-layered fenestrated sheets of wall material (numbered) and the small wall ingrowth papillae arising from the most recently deposited layer (darts). Single scale bar for **(A,B)** = 2.5 μm; for **(C)** = 5 μm; for **(D,E)** = 1 μm; for **(F)** = 0.25 μm; for **(G)** = 0.5 μm.

The ingrowth walls of TCs are generally one of two architectural types—reticulate or flange (Figures 1A,B respectively) or in a few instances both wall ingrowth morphologies occur in the same cell with reticulate ingrowths deposited on the flanges (Figure 1C). Formation of the ingrowth wall commences with deposition of a uniform wall layer against the original primary wall (Figure 1D). It is from this uniform wall that wall ingrowth papillae of the reticulate morphology (Figure 1D), and ribs or bands of the flange morphology arise. If ingrowth wall deposition is polarized, the uniform wall defines that polarity. In TCs with reticulate wall ingrowths, the extent of reticulation varies from a few discrete papillae (Figure 1E) to branched antler-like wall ingrowths (Figure 1F) and extensive labyrinths of fenestrated layers of wall material (Figure 1G) formed by a repeating sequence of papillate ingrowths branching and fusing to form a wall layer parallel to the original wall (Talbot et al., 2001). Flange ingrowths are deposited as ribs or bands of wall material arising from the underlying wall (Figure 1B). The flanges often extend evenly for the length of the cell (Figure 1C) but they may be concentrated toward the outer periclinal wall (Figure 1B) thus exhibit a degree of polarization.

This review focuses on TCs associated with phloem transport and in particular those located within phloem tissues. The review commences by identifying key apoplasmic steps in the phloem transport pathway as a prelude to addressing questions about broad evolutionary trends in relation to TC occurrence, their spatial relationships with other phloem cell types, their functional significance in the phloem transport pathways and whether signaling cascades known to initiate *trans*-differentiation to a TC morphology in developing seeds (Dibley et al., 2009; Zhou et al., 2010; Andriunas et al., 2011, 2012) may be involved in induction of cells within the phloem to a TC morphology.

## Key apoplasmic steps in the phloem transport pathway

In vascular plants, transport conduits of the phloem are sieve tubes (STs). These are comprised of sieve elements (SEs). At maturity, SEs contain a parietal cytoplasm and are joined end-to-end and interconnected through sieve pores in their shared transverse walls to form STs extending from source leaves to heterotrophic sink organs. In metaphloem, each SE is symplasmically coupled with, and metabolically dependent upon, a cytoplasmically-enriched companion cell (CC) to form a sieve element/companion cell (SE/CC) complex. For protophloem, SEs may, or may not, be accompanied by CCs. In all cases, SEs or SE/CC complexes are embedded in a matrix of phloem parenchyma cells.

**Figure 2:**
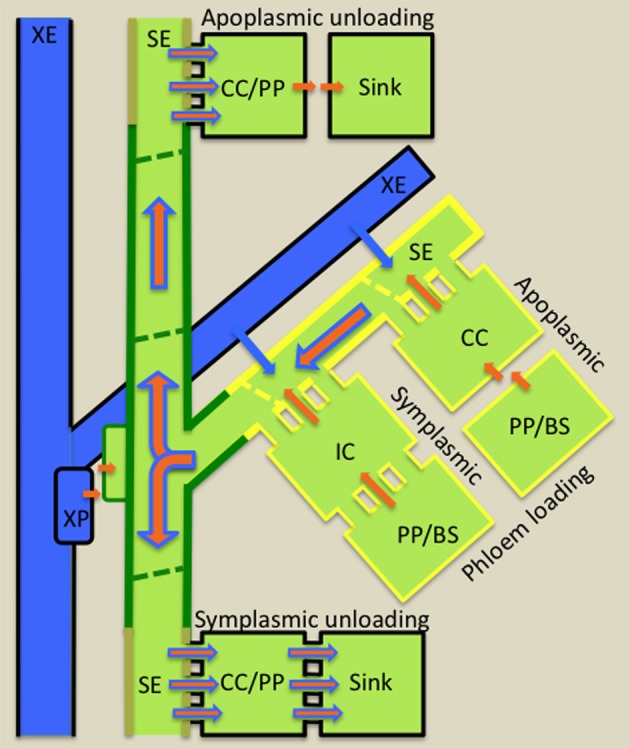
**Diagrammatic illustration of cellular pathways of water (blue arrows) and photoassimilate (brown arrows) loading into collection phloem (yellow border) in source leaves, of mineral nutrient exchange from xylem (blue) to transport phloem (green border) at nodes and of resource (water, mineral nutrients and photoassimilates) unloading from release phloem (kaki borders) in sinks.** Phloem loading mechanisms are defined according to cellular routes followed by photoassimilates moving from phloem parenchyma (PP) or bundle sheath (BS) cells to intermediary cells (IC; modified companion cells) or companion cells (CC) of collection phloem. Photoassimilate movement may occur from PP/BS cells through interconnecting plasmodesmata (symplasmic loading) to ICs or, in the absence of an adequate plasmodesmal connectivity, photoassimilates are effluxed from PP or BS cells into the cell wall matrix from which they are loaded into adjacent CCs (apoplasmic loading). Thereafter, for either loading mechanism, photoassimilates (primarily sugars) move symplasmically into adjacent sieve elements (SEs) of collection phloem. Accumulation of sugars to high concentrations in SEs causes an osmotic uptake of water from adjacent xylem elements (XE and see blue arrows) to generate large hydrostatic pressures (1–2 MPa). These drive a bulk flow of resources (brown superimposed on blue arrows) through the sieve tube system from source leaves to sinks. At nodes, mineral nutrients are extracted from XEs by xylem parenchyma (XP) cells and retrieved from the nodal apoplasm into transport phloem SEs. Resource exit from SEs of release phloem in sinks commonly follows a symplasmic route. Thereafter, further transport through the post-SE unloading pathway can continue through interconnecting plasmodesmata (symplasmic unloading) or, in certain sink types, a symplasmic discontinuity diverts resource flow through the intervening apoplasm (apoplasmic unloading—e.g., developing seeds).

Resources (nutrients and water) and developmental and defense molecules form the cargo transported over long distances through STs (Turgeon and Wolfe, 2009). ST fluxes of nutrients and water are extraordinary high, in the order of 555 g biomass m^−2^ ST cross-sectional area s^−1^ and 500 L m^−2^ ST cross-sectional area s^−1^, respectively (Patrick, 2012). To achieve ST axial fluxes of these magnitudes, the collection phloem (Figure 2) is organized into a dissected network of leaf minor veins to optimize loading of nutrients into SEs to high concentrations. Photosynthetically-produced sugars reach phloem/bundle sheath parenchyma cells of the collection phloem by transport through a symplasmic domain (a series of cytosols interconnected by plasmodesmata) linking them with adjacent photosynthetic mesophyll cells. Thereafter, sugars may continue to move through a symplasmic route into collection phloem SEs (symplasmic loading—Figure 2) via a specialized CC, intermediary cell (IC). Symplasmic loading is common amongst woody species and herbaceous eudicot species in which raffinose oligosaccharides (RFOs) are the principal transported sugar synthesized in ICs from sucrose imported from mesophyll cells (Rennie and Turgeon, 2009). Alternatively, sugars are released from phloem parenchyma cells into the shared cell wall space (apoplasm) with subsequent uptake into SE/CC complexes (apoplasmic loading—Figure 2). Apoplasmic loading is common amongst monocots and herbaceous eudicots in which sucrose or polyols are the primary transported sugar. However, there is emerging evidence that herbaceous symplasmic loaders also are capable of apoplasmic loading (Rennie and Turgeon, 2009; Batashev et al., 2013 this volume).

Photosynthetically-produced sugars form the major osmotica in collection phloem SEs contributing to an osmotic uptake of water that in turn creates high hydrostatic pressures of 1 to 2 MPa to develop in collection phloem SEs (Patrick, 2012). These hydrostatic pressure heads drive bulk flow of phloem sap through the transport phloem (Gould et al., 2005). The transport phloem (Figure 2) functions to retain phloem sap content by retrieval of solutes leaked to their surrounding apoplasms and by radial exchange with adjoining phloem parenchyma cells (Thorpe et al., 2005). These activities ensure that elevated hydrostatic pressures are maintained throughout the ST system (Patrick, 2013).

Nodal regions of stems represent a major site for xylem-phloem exchange of mineral nutrients and amino nitrogen compounds destined for developing shoot tips, fruits, and seeds. For instance, in white lupin seedlings, phloem sap is increasingly enriched in amino acids as it ascends acropetally as a result of xylem-to-phloem transfer at nodes (Layzell et al., 1981). The cellular pathway of resource transfer from xylem parenchyma cells to SEs in nodal regions is yet to be elucidated. However, it must depend upon apoplasmic phloem loading to sustain SE hydrostatic pressures that would be otherwise dissipated if loading were symplasmic (Figure 2). Thus, xylem-to-phloem transfer undoubtedly relies on amino acid and mineral ion transporters as exemplified by recent findings in Arabidopsis. For instance, RNAi knock-down of a xylem parenchyma localized amino acid permease, AAP6 (Okumoto et al., 2002), caused reductions in amino acid levels in phloem sap destined for developing siliques (Hunt et al., 2010). Similar responses were obtained with T-DNA knock-out lines of CCs expressing, AAP2, in which amino acids were diverted from reaching leaves in the xylem transpiration stream to siliques by delivery within the phloem (Zhang et al., 2010).

The release phloem, located in sinks, functions as a manifold from which phloem sap exits by bulk flow from SEs through plasmodesmata linking SEs or SE/CC complexes with surrounding phloem parenchyma cells (Figure 2 and see Patrick, 2013). Exit (phloem unloading) rates of resources are determined by hydraulic conductances of plasmodesmata linking SEs with phloem parenchyma cells (Patrick, 2013). In some sink types, including interfaces between generations and certain biotrophic relationships as well as sinks accumulating sugars to high concentrations, a symplasmic discontinuity is present at cellular sites located along their post-SE unloading pathways. These symplasmic discontinuities necessitate high fluxes of resources exchanged across plasma membranes to, and from, the sink apoplasm (Patrick, 1997).

Accepting that TCs function to facilitate resource exchange at symplasmic/apoplasmic interfaces (see Section Introduction), this brief analysis of phloem transport identifies a short list of candidate sites along the source-to-sink phloem path where cell types could be modified to a TC morphology. In the collection phloem, phloem parenchyma cells and CCs of apoplasmic loaders could be modified to a TC morphology to enhance nutrient release to, and uptake from, phloem apoplasms, respectively (Figure 2). Retrieval of phloem content leaked to phloem apoplasms as well as xylem-phloem and apoplasmic phloem-phloem exchange at nodes could benefit from TC function (Figure 2). Equally in sinks, TCs could be anticipated to function to support high membrane fluxes of resources to, and from, cells located at sites of symplasmic discontinuities located along post-SE unloading pathways (Figure 2). In all cases where transport to, and from, SEs is symplasmic, the requirement for TC function is circumvented.

## Broad evolutionary trends in relation to cellular location of transfer cells within phloem transport pathways

With the evolutionary advancement of vascularization, TCs are found located in close proximity to sieve and xylem elements throughout the vascular highway. As anticipated (see Section Key Apoplasmic Steps in the Phloem Transport Pathway), TCs are found in the collection phloem of leaf minor veins and the transport phloem of the major vein network of leaves, petioles, stems, rhizomes and roots. For the release phloem, they are located within post-SE unloading pathways at apoplasmic/symplasmic interfaces, for example, in developing seeds (Offler et al., 2003) and secretory glands (Pate and Gunning, 1972). However, unlike those of the collection and transport phloem, these TCs *trans*-differentiate from non-vascular cells.

### Collection phloem

The collection phloem (van Bel, 1993) is confined to the minor vein network in leaves (Figure 2) including cotyledons of germinating seed (Pate et al., 1970). Early evidence of a role for TCs in phloem loading is found in the gametophyte “leaf” of the moss *Polytrichum commune* in which a row of parenchyma cells extending the length of the “leaf” and located below the photosynthetic lamellae exhibit small papillate wall ingrowths (Scheirer, 1983). These deuter TCs are connected with the leptoids (rudimentary SEs) of the stem. While TCs are reported to occur in leaf sheaths and ligules of lycopods (e.g., Warmbrodt and Evert, 1979) and leaves of ferns (e.g., Warmbrodt and Evert, 1978) and several families (Taxaceae, Taxodiacese, and Pinaceae) of Gymnosperms (Gamalei, 1989), it is in herbaceous species of Angiosperms (Gamalei, 1991; van Bel and Gamalei, 1992; Davidson et al., 2011) that their occurrence becomes significant (and see Section Functional Role of Transfer Cells in Phloem Transport).

Two types of collection phloem cells—CCs (Gunning et al., 1968) and phloem parenchyma cells (Pate and Gunning, 1969) form TCs in Angiosperms. The ingrowth walls of these TCs are exclusively reticulate (see Figure 1E and Table S1). In CC/TCs (Type A TCs—Pate and Gunning, 1969), the ingrowth wall is not polarized although it is substantially reduced adjacent to SEs (e.g., *Pisum sativum*—Wimmers and Turgeon, 1991) relative to their interfaces with phloem parenchyma and bundle sheath cells. In contrast, in phloem parenchyma TCs (Type B TCs—Pate and Gunning, 1969), ingrowth walls are strongly polarized toward SEs (e.g., *Arabidopsis*—Haritatos et al., 2000) but have some ingrowths adjacent to CCs (e.g., *Ammobium alatum*—Gunning and Pate, 1969 and see Table S1). In a few species, bundle sheath cells develop an ingrowth wall where they interface with CCs (Pate and Gunning, 1972), but this has not been reported as a common occurrence.

For the Angiosperms, TCs of the eudicots have been the most widely researched in terms of their phylogeny. Classical surveys of leaf minor veins of hundreds of species (Pate and Gunning, 1969; Gamalei, 1989; Turgeon et al., 2001) have shown that approximately 40 percent of all eudicot genera have collection phloem TCs (see Pate and Gunning, 1969 data set presented in Table 1). The Compositae dominate these data as shown by their removal reducing this estimate to ca 20% (see Table 1). Significantly, the latter estimate corresponds with one based on the independently collected data set of Gamalei (1989) which does not include the Compositae (see Table 1). Interestingly, in the absence of the Compositae, Gamalei (1989) established that 58 percent of species with minor vein TCs belonged to only two of the 130 families surveyed—the Fabaceae and Asteraceae. A detailed survey of the Asteraceae (Batashev et al., 2013 this volume) has revealed that 22.5 percent of the 315 species surveyed have phloem TCs; a percentage that reflects that for eudicots minus the Compositae (Table 1). However, it is clear from subsequent phylogenetic analyses that the TC morphology has been derived multiple times during Angiosperm evolution (Turgeon et al., 2001). Indeed, in their analysis of 97 families, Davidson et al. (2011) reported 8.25 percent of families with minor vein TCs. Within a species, one or both CC/TCs and phloem parenchyma TCs occur in minor veins (e.g., CC/TCs alone—*Pisum sativum*—Wimmers and Turgeon, 1991, CC/TCs plus phloem parenchyma TCs—*Senecio vulgaris*—Browning and Gunning, 1979 or phloem parenchyma TCs alone—*Arabidopsis—*Haritatos et al., 2000, and see Table S1). Estimates of the distribution of these TC type options within genera surveyed by Pate and Gunning (1969) establish that CC/TCs alone is the dominant pattern (Table 1); a result also found at a family level with 69 percent of species of the Asteraceae (Batashev et al., 2013 this volume) having CC/TCs alone (Table 1). Conversely, the small (ca 1) percent of species with only phloem parenchyma TCs (Table 1) may well indicate a pronounced difference in the leaf vascular organization of the species in which this collection phloem TC pattern occurs. For example, for *Arabidopsis thaliana* that displays phloem parenchyma TCs alone, all veins, except the midvein and basal portions of the secondary veins, are regarded from a physiological perspective as a minor vein network (Haritatos et al., 2000). For the Compositae, however, both CC/TCs and phloem parenchyma TCs occur in 80 percent of genera (Table 1). This substantial investment in TCs within leaf minor veins by members of the Compositeae is consistent with their herbaceous habit (Turgeon et al., 2001) and high growth rates.

**Table 1:** **Percentage of Angiosperm genera with companion cell and phloem parenchyma transfer cells (TCs) in their minor veins**.

**Data source**	**Sub taxon/family**	**No of genera/species**	**Percent genera with TCs**	**Percent transfer cell type**		
				**CC**	**CC-PP**	**PP**
Pate and Gunning, 1969	Eudicot	3092^a^	43.8	25.8	16.6	1.3
	Eudicot minus Compositae^a^	2192^b^	21.0	15.8	4.2	1.1
Gamalei, 1989	Monocot	26^c^	15.4	ND	ND	ND
	Eudicot	393^c^	22.6	ND	ND	ND
Batashev et al., 2013	Asteraceae	315^c^	22.5	15.6	6.3	0.6

Data are derived from surveys by Pate and Gunning (1969), Gamalei (1989) and Batashev et al., this issue. CC, companion cell; CC-PP, companion cell–phloem parenchyma; PP, phloem parenchyma.

a Estimated from the total number of genera in each family using the proportion of the sampled genera with transfer cells.

b Compositae have been removed from the calculation described in ^*a*^ to provide data comparable to Gamalei (1989) that does not include this family in which all genera have transfer cells.

c Number of genera/species examined.

Phloem-associated TCs also *trans*-differentiate within transverse and third order longitudinal veins of the collection phloem of monocots, for example, in genera of the Zosteraceae, Liliaceae, Alliaceae, and Hemerocallidaceae (Gamalei, 1989 and see Table S1). However, there are relatively few reports of collection phloem TCs in monocots.

### Transport phloem

Transport phloem (Figure 2) is located in the major vein network of leaves and extends through petioles to the vascular systems of stems, rhizomes and roots. TCs associated with transport phloem are located in leaf major veins and petioles (Winter, 1982), within stem internodes (Kuo et al., 1980) and nodal complexes (Pate et al., 1970; Zee, 1972; Bushby and O'Brien, 1979), in corms (Cholewa and Griffith, 2004) and rhizomes (Yeung and Peterson, 1974) and at main/lateral root junctions (Letvenuka and Peterson, 1976; Ciamporova, 1993) (see Table S2). *Trans*-differentiation of parenchyma associated with xylem elements and CCs and parenchyma associated with SEs to a TC morphology occurs most strikingly in stem nodes (e.g., Gunning et al., 1970) consistent with a crucial role in redistribution of nutrients between the xylem and phloem for efficient distribution of resources (Gunning and Pate, 1974 and see Sections Key Apoplasmic Steps in the Phloem Transport Pathway and Functional Role of Transfer Cells in Phloem Transport).

The earliest definitive evidence of phloem-associated TCs in transport phloem is of TCs located at leaf gaps in the stem of the fern allie, *Equisetum arvense* (Hebant and Guillaume, 1983). In an evolutionary context, the absence of TCs in more primitive taxa reflects co-evolution of TC *trans*-differentiation within the transport vascular system and appearance of leaf gaps (where the leaf vascular system separates from that of the stem) with the development of the megaphyllous habit (leaves with a divided venation—Gunning et al., 1970). Stem TCs are reported, without distinction between those associated with the xylem or phloem, in ferns and gymnosperms being present in 69 and 50 percent of genera studied, respectively (Gunning et al., 1970 and see Table 2). This strikingly high occurrence is also evident in nodal regions of stems of Angiosperms with TCs present in 67 percent of genera of 58 families surveyed (Gunning et al., 1970 and see Table 2). The highest occurrence of genera was in eudicots (Table 2) and particularly in the Compositae, Leguminosae, Scrophulariaceae, and Umbelliferae. While these percentages must be considered with caution (Table 2 footnotes), when compared with the 22—30 percent occurrence of collection phloem TCs (Table 1), they underscore the functional importance of TCs in nodal regions of stems (see Section Key Apoplasmic Steps in the Phloem Transport Pathway). The significance of the difference between occurrence of TCs in collection and transport phloem is further emphasized by the observation that for the 34 families in which nodal TCs were recorded (Gunning et al., 1970), only 50 percent of the genera also had TCs in their minor veins.

**Table 2:** **Percentage of genera of Ferns, Gymnosperms and Angiosperms with nodal transfer cells**.

**Taxon/ sub taxon**	**No of genera**	**Percent genera with transfer cells**
Ferns	16	69
Gymnosperms	10	50
Angiosperms		
Monocot	13	39
Eudicot	116	70

Data are derived from a survey of 61 families by Gunning et al. (1970) in which no distinction is made between xylem and phloem transfer cells. In their survey Gunning and co-workers emphasized that “negative results must be viewed with caution since in certain cases the zone of transfer cell formation” (within the node) “is known to be extremely localized.”

There are few reports of phloem TCs in major leaf veins and the vascular systems of petioles and roots (Table S2). However, in *Trifolium alexandrinum*, phloem parenchyma TCs associated with petiolar and major leaf veins play a central role in intraveinal recycling of sodium ions entering leaves in the transpiration stream (Winter, 1982).

Nodal xylem parenchyma TCs may have either a reticulate morphology with their ingrowth walls polarized to a xylem element (e.g., *Tradescantia virginiana—*Gunning et al., 1970) or display a flange morphology (e.g., *Triticum aestivum—*Bushby and O'Brien, 1979), the latter being particularly prevalent in monocots (and see Section Evolution and Wall Ingrowth Architecture). In contrast, ingrowth walls of nodal phloem parenchyma TCs are always reticulate and polarized to SEs (e.g., *Pisum sativum*—Newcomb and Peterson, 1979 and see Table S2). Within nodal regions, xylem parenchyma TCs occur most prominently in association with departing foliar traces (Pate and Gunning, 1972; Zee, 1974; Bushby and O'Brien, 1979) but may extend beyond the node (Kuo et al., 1980). The phloem parenchyma TCs, while reported to line the flanks of leaf traces (Pate and Gunning, 1972) are most frequently associated with vein anastomoses running longitudinally across the node and connecting axial veins with leaf traces often one or two internodes below the position of emergence of the leaf trace (e.g., in nodes of bamboo culms—Zee, 1974). In wheat stems, phloem parenchyma TCs occur in pith bundles that link leaves in all mature nodes (Bushby and O'Brien, 1979). These TCs appear to play a crucial role during early leaf development by differentiating in the mid vein of an older leaf that supplies resources to the next expanding leaf through nodal bridging strands. Similarly, in many eudicot species, nodal phloem TCs play a role in nutrient distribution early in plant development. For instance, phloem parenchyma TCs differentiate in the hypocotyl and cotyledons within 3 days of germination, in step with development of protophloem, to deliver nutrients to the elongating plumule (Pate et al., 1970) and, in subsequent nodes of the developing stem, they are associated with leaf traces.

As for the transport phloem in leaves, there are few reports of TCs associated with transport phloem in roots. However, both xylem and phloem parenchyma cells have been reported to *trans*-differentiate to a TC morphology where lateral roots arise from the main root axis. In these locations, TCs occur in the main root, the connective vascular system and the base of the emerging lateral root (e.g., *Hieracium florentinum*—Letvenuka and Peterson, 1976, *Helianthus spp*—Ciamporova, 1993). In the specialized roots of the seagrass *Zostera capensis*, CCs as well as phloem and xylem parenchyma cells are modified to form TCs (Barnabas and Arnott, 1987).

### Release phloem and post-sieve element unloading pathway

TCs associated with the release phloem *trans*-differentiate from non-vascular cells (Figure 2). In developing reproductive organs of many genera (e.g., *Phaseolus, Vicia, Lilium*) TCs occur at a number of locations during embryogenesis and in the pre-storage and storage phases of seed development (Gunning and Pate, 1974; Offler et al., 2003). Their most common location at the interface between generations, where apoplasmic exchange of nutrients is mandatory, has a long evolutionary history commencing in the algae and extending to the Angiosperms. The first evidence of TCs at this location is in the Characean alga *Coleochaete orbicularis* (Graham and Wilcox, 1983). In this species the single thallus cell that becomes specialized for reproduction and acts as a zygote ultimately producing motile zoospores, is surrounded by a ring of thallus TCs. Interestingly, the reticulate wall ingrowths of these TCs are polarized to the thallus/zygote interface, a feature retained for TCs in this location in all taxonomic groups. In non-vascular land plants, (hornworts, liverworts, mosses), fern allies (horsetails and lycopods) and ferns, TC *trans*-differentiation commonly occurs in cells bordering the gametophyte/sporophyte interface (see Table S3). These TCs form in one or both generations with their reticulate ingrowth walls polarized to the interface. An analysis of 18 genera (Table 3) highlights that, within the Bryophytes, TCs almost exclusively occur in tissues of both generations. An example where TCs are found only in the gametophyte is the hornwort *Anthroceros laevis* (Gunning and Pate, 1969) but such a situation is apparently very rare (Table 3). In the lycopod, *Lycopodium appressum* (Peterson and Whittier, 1991) and the ferns, *Adiantum cappillus-veneris and Polypodium vulgare* (Gunning and Pate, 1969), TCs are found in both the foot of the developing sporophyte and the gametophyte tissue in which it is embedded adding further evidence to support the pattern of formation of TCs in both generations.

**Table 3:** **Location of transfer cells at the interface between generations in Bryophytes and developing seeds of Angiosperms during their storage phase of growth**.

**Taxonomic group**	**No of genera**	**Percent genera with transfer cells**		
**Bryophytes**		**Gameto (G)**	**Sporo (S)**	**G + S**
Hornworts	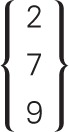			
Liverworts		5	5	89
Mosses				
**Angiosperms**		**Maternal**	**Filial**	**M + F**
Monocot	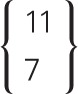			
Eudicot		16	56	28

Data are percent of genera with transfer cells in gametophyte and sporophyte or both tissues at this interface in Bryophytes and similarly for maternal and filial tissues of developing seeds of Angiosperms.

Data sources (for full references see reference list of supplemental data):

Bryophytes:

Alfayate et al. (2000), Carafa et al. (2003), Gambardella (1987), Gambardella and Ligrone (1987), Ligrone and Duckett (2011), Ligrone et al. (1982), Ligrone and Renzagnia (1990), Peterson and Whittier (1991), Yip and Rushing (1999).

Angiosperms—Monocots:

Cochrane and Duffus (1980), Felker and Shannon (1980), Fussell and Dwarte (1980), Hoshikawa (1984), Kuo et al. (1990), Rost et al (1984), Wada and Maeda (1981), Wang et al. (2012), Weschke et al. (2000), Zee and O'Brien (1971), Zheng and Wang, Z. (2011).

Angiosperms—Eudicots:

Farley et al. (2000), Gori and Sarfatti (1970), Gori (1987), Greenwood et al. (2005), Newcomb (1978), Offler et al. (1989), Pugh et al. (2010), Tegeder et al. (1999), Wise and Juncosa (1989).

In hornworts, liverworts, fern allies and ferns, the transport path to, and from, the generation interface is only a few cells. In mosses, however, with their upright gametophyte supporting a sporophyte with a seta (stalk) supporting the spore-bearing capsule, there are extensive symplasmic pathways to, and from, TCs bordering the gametophyte/sporophyte interface (e.g., *Funaria hygrometrica*—Browning and Gunning, 1979; *Timmiella barbuloides*—Ligrone et al., 1982). This same spatial relationship of a symplasmic pathway culminating, or commencing, with TCs is preserved in release phloem of developing Angiosperm seeds during their storage phase of growth (Figure 2). In developing seeds of both monocots and eudicots, a symplasmic post-SE pathway may culminate in TCs at the maternal/filial interface (e.g., seed coat TCs of cotton—Pugh et al., 2010 and see Figure 1C) and TCs may begin an equivalent symplasmic pathway in the filial tissue (e.g., basal endosperm transfer cells in maize—Davis et al., 1990 and see Figure 1B). For seeds, the percentage of genera exhibiting only one or both maternal and filial TCs differs markedly from that of the Bryophtes with a clear shift (53 percent) to a filial only pattern (Table 3). The genera retaining TCs in tissues of both generations are cereals and large-seeded grain legumes (e.g., *Triticum aestivum*—Wang et al., 1994, 1995 and *Vicia faba*—Offler et al., 1989). With the exception of nucellar projection TCs in wheat and barley that project into the endosperm cavity (Wang et al., 1994; Cochrane and Duffus, 1980), the ingrowth walls of these TCs are polarized toward the maternal/filial interface maintaining a common pattern first evident in the algae.

### Phloem invaders

A more recent evolutionary development has been invasion of the transport phloem by opportunistic organisms. Pronounced examples include invasion of leaf/stem transport phloem by hoppers (Ecale-Zhou and Backus, 1999), the stem and root transport phloem by vascular holoparasites (Hibberd and Jeschke, 2001), root transport phloem by nitrogen-fixing bacteria forming nodules (Pate and Gunning, 1972) and root knot nematodes (Barcala et al., 2010 and for more details see Section Invaders Induce Transfer Cell Formation in Transport Phloem). In each case, the transport phloem may be morphed into a release role to facilitate the requirements of the invader that includes the formation of TCs. A common theme emerges for the “modus operandi” of biotic invaders of the vascular system. For each type of invading organism, some members have exploited establishing direct lumen-to-lumen connection with xylem elements or a symplasmic connection with phloem SEs, while others induce xylem or phloem TCs to facilitate apoplasmic exchange. These options are evident between species of stem and root parasites (Hibberd and Jeschke, 2001), root-knot and cyst nematodes (Hoth et al., 2008) and the nodules of nitrogen-fixing bacteria (Pate and Gunning, 1972).

### Evolution and wall ingrowth architecture

Reticulate wall ingrowth architecture (Figure 1A) is considered to be the primitive state being characteristic of all TCs in algae, fungi, non-vascular land plants, fern allies and ferns (Suppl Figure 1, Offler et al., 2003). It has been retained throughout evolution and remains dominant in both monocots and eudicots of Angiosperms with 94 percent of 274 observations of TCs exhibiting this architectural type (data from Offler et al., 2003, Suppl Table 1). In contrast, flange architecture (Figure 1B) is first reported for gametophyte cells adjacent to the embryo in the Gymnosperm *Podocarpus henkelii* (van Staden et al., 1976). The small (6 percent) of TCs with a flange morphology recorded for Angiosperms are largely associated with the xylem and are most prevalent in monocots (25 percent of 51 observations of TCs in monocots) with only limited reports (e.g., Figure 1C) of this wall ingrowth architecture occurring in TCs of nodal regions and developing seeds of eudicots.

We can only speculate on the implications for membrane transport of these two types of wall ingrowth architecture. Reticulate architecture exhibits a wide variation in extent of wall ingrowth deposition that does not follow an evolutionary trend. For example, the thallus TCs of the characean alga *Coleochaete orbicularis* (Graham and Wilcox, 1983) have long papillate wall ingrowths and sporophyte foot TCs of the lycopod, *Lycopodium appressum* (Peterson and Whittier, 1991) have an ingrowth wall composed of multiple fenestrated layers of wall material. In contrast, seed coat TCs of the Angiosperm, *V. faba*, are characterized by a few small papillate ingrowths (Offler and Patrick, 1993 and see Figure 1E). Rather, the extent of wall ingrowth deposition may well be linked to location and the consequent demand for nutrient exchange. For example, in CC/TCs of minor veins (*Pisum sativum*—Wimmers and Turgeon, 1991) there is limited development of wall ingrowths. This contrasts with the multiple fenestrated layers of an extensive ingrowth wall of epidermal cotyledon TCs of *V. faba* (Talbot et al., 2001). Flange morphology provides a lesser capacity to amplify plasma membrane surface areas and part of the role of these TCs may be for mechanical support. This suggestion is supported by seed coat TCs of cotton in which reticulate ingrowths are deposited on the flanges (Figure 1C). For these TCs estimates of the contribution of flange and reticulate ingrowths to total membrane surface area per μm^2^ of wall of a cultivated tetraploid cotton with its two diploid progenitors (Pugh et al., 2010) established that reticulate ingrowths contributed 70—74 percent of total membrane surface area indicating a minor role for flange ingrowths in providing nutrient exchange capacity.

## Functional role of transfer cells in phloem transport

In Angiosperms, TCs may *trans*-differentiate from phloem parenchyma or CCs of collection and transport phloem. In contrast, TCs are absent from release phloem *per se* (see Section Release Phloem and Post-Sieve Element Unloading Pathway and Figure 2). The spatial orientation of their ingrowth walls in relation to surrounding cell types provides some hints as to their function in canalizing cell-to-cell transport of resources into, or from, the phloem. Contributions of TC cohorts to rates of intercellular transport are inferred from the degree to which the architecture of their wall ingrowths amplifies surface areas of their plasma membranes (see Section Evolution and Wall Ingrowth Architecture and Figure 1) to increase transporter numbers and hence membrane transport activity per cell. In this context, modification of cells located along phloem transport pathways into TC morphologies would be expected to enhance their solute and water fluxes and hence rates of phloem transport. However, as reviewed below, current evidence in support of this proposition is scarce and fragmentary for collection phloem, transport phloem and post-SE unloading pathways from release phloem. This situation equally applies to plant host/biotroph interfaces at which TCs form proximal to, or within, the phloem (see Section Phloem Invaders).

### Collection phloem

In leaf architectures for 40 percent of all herbaceous eudicots surveyed (Pate and Gunning, 1972; Gamalei, 1989; Turgeon et al., 2001), in which there is minimal symplasmic connectivity between phloem parenchyma and CCs, one or both of these cell types can be modified to a TC morphology with CCs being the most common (see Table 1 and associated text). This conclusion also applies to mixed loading species (see Batashev et al., 2013 this volume and Table 1). Spatial organization of ingrowth walls in phloem parenchyma and CCs modified to a TC morphology are consistent with funneling resources to SEs. For instance, the wall ingrowths of CC/TCs are substantially reduced from shared walls with adjoining SEs (see Section Collection Phloem and Table S1). In contrast, wall ingrowths of phloem parenchyma TCs tend to occur proximal (e.g., *Senecio vulgaris*) or solely adjacent (e.g., *Arabidopsis thaliana*) to SEs (e.g., see Amiard et al., 2005; Table S1). This arrangement may favor an enhanced preferential flow of resources toward SEs to supplement resource efflux occurring in non-ingrowth wall regions of bundle sheath and phloem parenchyma cells into abutting regions of wall ingrowths in adjacent CCs.

Sucrose or polyols are the major osmotic species of apoplasmic phloem loaders contributing to elevating SE/CC complex hydrostatic pressures by osmotic water uptake across their plasma membranes, facilitated by aquaporins, to drive bulk flow of resources through the phloem from source to sink (Figure 2; Patrick, 2012). Surprisingly, there is scant published evidence for the presence of sugar transporters or aquaporins on plasma membranes of phloem parenchyma or CCs modified to a TC morphology. The exception is members of a recently discovered sub-family of plasma membrane sucrose facilitators, SWEETs. These transporters localize to plasma membranes of non-SE/CC phloem cells of Arabidopsis leaf minor veins that are most likely phloem parenchyma TCs (Chen et al., 2012). Phenotypes of double T-DNA knock-out mutants of *AtSWEET11* and *AtSWEET12* demonstrated that SWEETs function to release sucrose from putative phloem parenchyma TCs for subsequent uptake into SE/CC complexes and export from leaves (Chen et al., 2012). Consistent with energizing sucrose/proton symport loading of SE/CC complexes, H^+^-ATPases have been cytochemically localized to plasma membranes lining wall ingrowth regions in CC/TCs of *Pisum sativum* (Bentwood and Cronshaw, 1978) and *V. faba* (Bouché-Pillon et al., 1994). However, to our knowledge, sugar transporters and aquaporins are yet to be localized to CCs with wall ingrowths. Nevertheless, there is good evidence for the presence of sucrose transporters, and, to a lesser extent, aquaporins, on plasma membranes of ordinary SE/CC complexes (Tegeder et al., 2012). Assuming that this applies to CCs modified to a TC morphology, the following analysis is undertaken.

Sugar concentrations presenting to plasma membrane transporters of phloem parenchyma cells for efflux, and of SE/CC complexes for influx, are of magnitudes that likely saturate transporter activity (Patrick, 2012). Thus, one option for upregulating rates of phloem loading is to increase numbers of sugar and water transporters per cell through amplifying plasma membrane surface areas of phloem parenchyma cells or SE/CC complexes or both of these cell assemblages. This scenario has been explored by two independent approaches in which the impact of altering the extent, but not induction, of wall ingrowth formation on phloem loading/export was evaluated. The first approach exploited the observation that wall ingrowth formation in collection phloem cells is positively responsive to light intensity incident on the leaf surface (Gunning et al., 1968). Here *Pisum sativum* plants were raised under two levels of photosynthetically-active radiation (200 vs. 1000 μmol. m^−2^ s^−1^). The high light growth conditions evinced a 50 percent increase in plasma membrane surface areas of collection phloem SE/CC complexes attributable to a more extensive development of wall ingrowths in their CC/TCs. The increased plasma membrane surface area of CC/TCs was accompanied by a comparable relative increase in rates of phloem loading (Wimmers and Turgeon, 1991). This study was extended to include Arabidopsis with phloem parenchyma TCs and *Senecio vulgaris* in which both phloem parenchyma and CCs are modified to a TC morphology. Irrespective of the anatomical configuration of TCs, the extent of their wall ingrowths, and hence presumably phloem transport rates, responded positively to incident light intensity (Amiard et al., 2007). The second approach to link wall ingrowth deposition with phloem loading capacity of collection phloem comes from studies of Arabidopsis vitamin E (*vte*) biosynthetic mutants and, in particular, *vte2*. Raised under cold conditions, the *vte2* mutant exhibits a biochemical phenotype of excessive carbohydrate accumulation in source, but not sink, leaves consistent with attenuation of phloem loading and export as verified by ^14^C-photoassimilate export rates from excised leaves (Maeda et al., 2006). More importantly, upon transfer to low temperature conditions, onset of slowed rates of photoassimilate export coincided with callose being selectively deposited in wall ingrowth regions of phloem parenchyma TCs (Maeda et al., 2006). Interestingly, exposure to cold causes an enhanced development of wall ingrowths in wild type phloem parenchyma TCs that undoubtedly ameliorates low temperature impacts on rates of resource export from leaves (Maeda et al., 2006). Together, responses to light intensity and low temperature provide persuasive evidence that TCs, through an amplified surface area of plasma membrane, play a central role in phloem loading. The formation of an ingrowth wall in phloem parenchyma TCs also has been linked with a biotic stress response to limit spread of fungal spores and bacteria through the phloem (Amiard et al., 2007; Demming-Adams et al., 2013).

### Transport phloem

As a generalization most xylem and phloem parenchyma TCs of the transport phloem are localized to nodal regions of stems (see Section Transport Phloem) and sites of emergance of lateral roots (e.g., Ciamporova, 1993) with xylem parenchyma TCs tending to be the most ubiquitous and conspicuous of the two TC types (see Section Transport Phloem). These TCs are conjectured to function in xylem-phloem and phloem-phloem exchange of resources moved to acropetally-located sinks (see Section Key Apoplasmic Steps in the Phloem Transport Pathway). The only available data to support this contention applies to xylem-phloem transfer in flag leaf nodes of graminaceous species. For instance, in rice flag leaf nodes, xylem parenchyma TCs, located in internodal and leaf-trace vascular bundles, are interlinked to diffuse and anastomosing vascular bundles with the diffuse vascular bundles serving the panicle (Zee, 1972). In this context, a plasma membrane silicon transporter, Lsi6, localizes to nodal xylem parenchyma TCs (Yamaji and Ma, 2009) and a broad specificity cation effluxer localizes to phloem parenchyma cells of diffuse bundles (Uraguchi et al., 2011). The latter is consistent with an apoplasmic loading mechanism for their SE/CC complexes. Knockout and knockdown, respectively of these transporters resulted in significant reductions of silicon and cadmium reaching the panicles (Yamaji and Ma, 2009; Uraguchi et al., 2011). These findings are consistent with xylem parenchyma TCs withdrawing these elements from the transpiration stream followed by symplasmic delivery to phloem where an apoplasmic loading step occurs at the phloem parenchyma cell and SE/CC interface. Rice nodes lack phloem parenchyma TCs that may perform this function in other species in which these TCs occur (see Section Transport Phloem).

Coupled with xylem-to-phloem transport of ions is the question of shoot-delivered sodium recycled to roots through the phloem, the significance of which as a salt tolerance mechanism is unresolved (Munns and Tester, 2008). Sodium delivery to shoots is regulated by the activity of xylem parenchyma-localized AtHKT1, or its orthologues in rice and wheat, that functions to withdraw sodium from the transpiration stream (Horie et al., 2009). Recently, a CC expressed metal binding protein, NaKR1, was found to reduce sodium accumulation in leaves by upregulating phloem loading and export of sodium (Tian et al., 2010). The NaKR1 function maps onto earlier findings that exposure of *Trifolium alexandrium* (Winter, 1982) and *Medicago sativa* (Boughanmi et al., 2003), to elevated sodium levels induced CCs of leaf transport phloem to undergo *trans*-differentiation to a TC morphology. In addition, observed salt-induced increases in levels of unmethylated pectins, fucosylated xyloglucans, and arabinogalactans in wall ingrowths of CC/TCs might act to buffer free sodium levels in leaf apoplasms (Boughanmi et al., 2010).

### Release phloem and post-sieve element unloading pathways

Release phloem characteristically unloads symplasmically (Patrick, 2012). Therefore, there is no surprise that TCs are absent from release phloem. However, in a cohort of monocot and eudicot species, post-SE unloading pathways of developing seeds contain TCs positioned at their maternal/filial interfaces (see Section Release Phloem and Post-Sieve Element Unloading Pathway; Figures 1 and 2, Table S3). In general, their ingrowth walls are polarized in the direction of resource flow from maternal to filial tissues and, within these regions, their plasma membranes are enriched in membrane transporters (Zhang et al., 2007). Notable amongst these transporters are sucrose facilitators in maternal TCs of eudicots and monocots (Zhang et al., 2007) that could well include the recently discovered SWEETS (Chen et al., 2012). Physiological evidence suggests that sucrose release from legume seed coats is also energized by a proton/antiport mechanism but the encoding gene(s) are yet to be identified (Zhang et al., 2007). Release of amino nitrogen compounds may occur through ion channels (Zhang et al., 2007). Consistent with a role for TCs in regulating water release from maternal seed tissues (see Figure 2), a recent transcriptome analysis detected strong expression of aquaporins (both PIPS and TIPS) in nucellar projection TCs of barley grains (Thiel et al., 2008). For filial seed tissues, sucrose and amino acid uptake is energized by proton-coupled symporters expressed in epidermal TCs of legume cotyledons and modified aleurone cells of cereal grains (Zhang et al., 2007; Thiel et al., 2008, 2012). Sucrose symporters, located in filial TCs in developing seeds of pea (Rosche et al., 2002) and wheat (Weichert et al., 2010), have been shown to play a key role in the carbon economy of filial storage tissues. Similar functions are deduced for the maternally-located TCs (Zhang et al., 2007). As described below, attenuating formation of ingrowth walls in filial TCs exerts an even greater impact on development and biomass of filial tissues.

Interploidy crosses in maize were found to compromise wall ingrowth formation in the basal endosperm TC layer (BETL) of developing kernels. The resulting shrunken kernels are attributed to an attenuated resource delivery to the endosperm through reduced wall ingrowth formation in the TC layer (Charlton et al., 1995). These observations have been followed up using a suite of maize mutants including *reduced grain filling1 (rgf1*—Maitz et al., 2000), *globby1* (*glol-1*—Costa et al., 2003), *baseless1* (Gutiérrez-Marcos et al., 2006), *empty pericarp4* (*emp4*—Gutiérrez-Marcos et al., 2007) and miniature1 (*mn1*—Kang et al., 2009). All these mutants negatively impact early development of the basal endosperm TCs and exhibit a shrunken kernel phenotype. The causal relationship between attenuated wall ingrowth formation and the shrunken phenotype is clearest for the *mn1* mutant encoding a cell wall invertase, INCW2, which is expressed specifically in the basal endosperm TCs and impacts their early development (Kang et al., 2009). However, the most compelling evidence for a causal relationship between wall ingrowth development and resource supply to the developing endosperm, is the shrunken kernel phenotype resulting from RNAi knockdown of *Maternally expressed gene 1* (*Meg1*—Costa et al., 2012). *Meg1* encodes a small cysteine-rich peptide that specifically localizes to plasma membranes of differentiating endosperm TCs and regulates expression of TC specific genes, including INCW2 (Costa et al., 2012). TCs appear to serve a similar function in resource loading of eudicot seeds. For instance, a pea seed mutant, E2748, blocks *trans*-differentiation of abaxial cotyledon epidermal cells to a TC morphology and negatively impacts embryo development (Borisjuk et al., 2002). In addition, the positive relationship between plasma membrane amplification by wall ingrowths and seed biomass yield was found to be upheld in a comparative analysis of cultivated tetraploid cotton with its two diploid progenitors (Pugh et al., 2010).

### Invaders induce transfer cell formation in transport phloem

Invasion of stems by leafhoppers (Ecale-Zhou and Backus, 1999), of stem and root vascular systems by holoparasites (Hibberd and Jeschke, 2001) and of root cortical cells by nitrogen-fixing bacteria forming nodules (Pate and Gunning, 1972), can cause TCs to form in (Ecale-Zhou and Backus, 1999), or adjacent to (Pate and Gunning, 1972; Hibberd and Jeschke, 2001), the transport phloem. In contrast, root knot nematodes invade provascular cells or partially differentiated tracheary elements located in root tips and induce formation of giant cells (Barcala et al., 2010) with wall ingrowths abutting both SEs and xylem elements (Hoth et al., 2008). For root nodules and nematode giant cells, TC formation is accompanied by differentiation of additional phloem and xylem that link nodules or giant cells with the root vascular system (Pate and Gunning, 1972; Hoth et al., 2008). The phloem component of the additional vasculature functions as release phloem to supply nutrients and water to the enclosed root nodules (Complainville et al., 2003) or nematode giant cells (Hoth et al., 2008).

The question is what function(s) do TCs perform at host/biotroph interfaces. For leafhoppers, development of wall ingrowths in host transport phloem CCs could be a defense response to limit loss of transport phloem content to the surrounding stem apoplasm and hence the invading leafhoppers (Ecale-Zhou and Backus, 1999). TC formation in haustorial “absorbing hyphae” of holoparasites abutting host SEs (Dörr, 1990) or pericycle cells abutting root nodule SE/CCs (Pate and Gunning, 1972) co-occurs with developing a symplasmic domain, competent in solute and macromolecular transport, linking the holoparasite (Birschwilks et al., 2006, 2007) or nitrogen-fixing bacteroids (Complainville et al., 2003) with transport phloem SEs. Thus, at best, TC transport function at these biotrophic interfaces is reduced to a supplementary role in delivering phloem content to the biotroph that would be expected to predominantly flow through a post-SE symplasmic route (Patrick, 2012). In contrast, nematode giant cells are symplasmically isolated from adjoining release phloem SEs that retain their nuclei and lack CCs (Hoth et al., 2008). Consistent with symplasmic isolation, a significant upregulation in transporter expression occurs during root knot infestation of Arabidopsis (Hammes et al., 2005, 2006; Barcala et al., 2010). In terms of resource allocation, included amongst the population of upregulated transporters expressed in root knot tissue were aquaporins (PIPs and TIPs) linked with early volume growth of giant cells (Barcala et al., 2010), an array of amino acid transporters (Hammes et al., 2005, 2006) and a sucrose transporter, AtSUC1 (Hammes et al., 2005). Consistent with a key transport function, nematode infection was considerably compromised in an amino acid permease (AAP) double mutant, *aap3* and *aap6* (Marella et al., 2013).

## Signals regulating transfer cell *trans*-differentiation—developing seeds show the way

Despite the key physiological significance of TCs in phloem transport of resources (see Section Functional Role of Transfer Cells in Phloem Transport), the regulatory mechanisms directing assembly of their ingrowth walls are poorly understood (McCurdy et al., 2008). A major impediment to progressing understanding of this developmental phenomenon is that most TCs are deeply embedded within plant tissues and occur in small numbers (Offler et al., 2003). An essential ingredient to bridge this impasse is an experimental system that provides access to a large population of developmentally-uncommitted cells undergoing synchronous *trans*-differentiation to a TC morphology. As described below, adaxial epidermal cells of cultured cotyledons of *V. faba* (Offler et al., 1997) meet these requirements.

**Figure 3:**
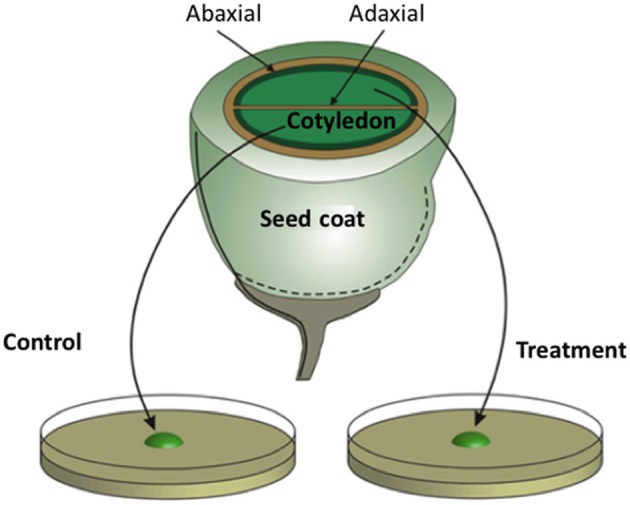
**Schematic of a transfer cell inductive system using *Vicia faba* cotyledons.** An equatorial view of a *V. faba* seed sliced transversely in half. A maternal seed coat encloses two large cotyledons. At the maternal/filial interface, thin-walled parenchyma cells of the seed coat and abaxial epidermal cells of the cotyledons (solid dark green lines) *trans*-differentiate to a transfer cell morphology. In contrast, adaxial epidermal cells of the cotyledons do not *trans*-differentiate to a transfer cell morphology. Upon surgical removal of cotyledons from their enclosing seed coats and transfer to a MS medium, cotyledon adaxial epidermal cells undergo a synchronous *trans*-differentiation to transfer cells that are structurally and functionally identical to their abaxial counterparts. Sister cotyledons of each seed are randomly allocated to MS medium alone or a MS medium carrying a pharmacological agent that interferes with developmental signals or wall building machinery.

Unlike their abaxial counterparts, adaxial epidermal cells of *V. faba* cotyledons do not form a TC morphology *in planta* (Offler et al., 1997; see Figure 3). However, when isolated *V. faba* cotyledons are cultured on nutrient agar, their adaxial epidermal cells are rapidly (h) induced to form wall ingrowths on their outer periclinal walls (Wardini et al., 2007). This system is unique in providing access to a large population of uncommitted epidermal cells (several thousand) that, upon exposure to culture conditions, are synchronously induced to *trans*-differentiate to a TC morphology across a defined temporal window (Wardini et al., 2007). Developmental synchronicity of the epidermal cell population permits temporal separation of uniform wall deposition from wall ingrowth assembly (see Figure 1D). The capacity to peel the induced adaxial epidermis from cotyledons provides large quantities of material for cell-specific gene expression profiling at specified stages of ingrowth wall formation (e.g., Dibley et al., 2009). Exposure of sister cotyledons to pharmacological blockades (Figure 3) offers an experimental opportunity to investigate regulation of *trans*-differentiation events through the inductive signaling sequence (Zhou et al., 2010; Andriunas et al., 2011, 2012; Xia et al., 2012). Statistically robust quantification of ingrowth wall formation responses to pharmacological treatments can be derived from scoring large populations of synchronously *trans*-differentiating adaxial epidermal cells for presence of wall ingrowths in epidermal peels prepared by a dry-cleave method to visualize cytoplasmic faces of their outer periclinal walls by SEM/FESEM (Talbot et al., 2001). Live and real-time visualization of the spatial organization of regulatory proteins and their signals directing ingrowth wall formation in epidermal cells is available through confocal imaging of thick (100 μm) tissue sections of cotyledons exposed to specified treatments. Importantly, culture-induced adaxial epidermal transfer cells mimic their *in planta* abaxial counterparts in terms of wall ingrowth morphology (Talbot et al., 2001) and transport function (Farley et al., 2000). Thus, the *V. faba* cotyledon culture system (Offler et al., 1997) offers a validated experimental platform from which to deduce developmental processes orchestrating *trans*-differentiation to a TC morphology *in planta*.

**Figure 4:**
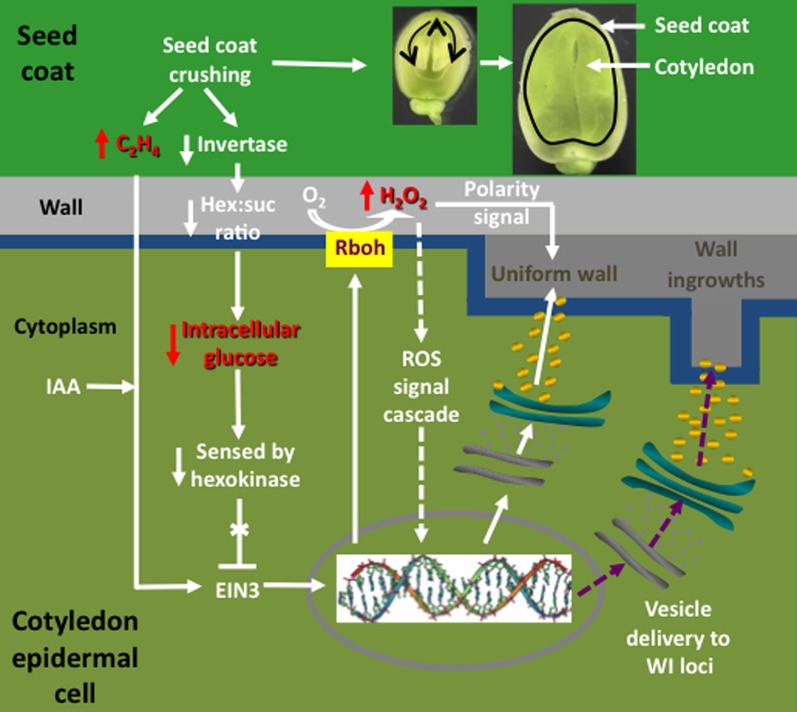
**Interrelationships between signals and signal cascades leading to induction, organization, and construction of polarized ingrowth walls in epidermal transfer cells of *Vicia faba* cotyledons.** In cultured cotyledons, a combination of declining intracellular glucose levels and an auxin (IAA) regulated burst in ethylene production, initiates ingrowth wall formation within adaxial epidermal cells. The glucose/ethylene sequence is reproduced *in planta* and downstream events are deduced from findings obtained using cultured cotyledons. Prior to the onset of cotyledon growth, an extracellular invertase, localized to inner cells of the seed coat (dark green), hydrolyzes sucrose and the glucose product, sensed by a cotyledon epidermal cell (light green) hexokinase, blocks an ethylene signal cascade by down regulating ethylene insensitive 3 (EIN3), a key ethylene signal cascade transcription factor. Upon initiating expansion growth, the cotyledons crush inner cells of the seed coat (see images of seeds cut through their longitudinal plan illustrating cotyledon growth at two stages). Cell crushing results in a loss of extracellular invertase activity and hence the glucose signal. This is accompanied by an amplified wound-induced ethylene (C_2_H_4_) signal cascade, mediated through EIN3, driving expression of respiratory burst oxidases (Rbohs), trafficked to portions of the plasma membrane (blue) that line the outer periclinal walls of cotyledon epidermal cells. The Rbohs catalyze production of extracellular reactive oxygen species (ROS) that are further reduced to form an extracellular hydrogen peroxide (H_2_O_2_) signal that serves two known functions. These are activating expression of cell wall building machinery through an unknown signal cascade (arrows with broken shafts) and serving as a positional signal directing construction of the uniform wall (dark gray) polarized to the outer periclinal walls (light gray) of the cotyledon epidermal cells. How the polarized wall construction subsequently is constrained to loci from which wall ingrowth papillae arise remains to be determined (purple arrows with broken shafts) but likely involves directional control by cytosolic calcium signals.

Early experiments utilizing transcriptome profiling found that an enhanced proportion of genes, selectively induced in adaxial epidermal cells undergoing *trans*-differentiation to a TC morphology, carry auxin- and ethylene-responsive elements in promoter regions of their orthologous genes (Dibley et al., 2009), consistent with coordinated regulation by auxin and ethylene (Swarup et al., 2002). A role for auxin inducing adaxial epidermal cells to form TCs was confirmed utilizing auxin transport and synthesis inhibitors (Dibley et al., 2009; Zhou et al., 2010 and see Figure 4). Ethylene was shown to act as a regulatory signal initiating and sustaining *trans*-differentiation to a TC morphology (Zhou et al., 2010). Temporal and spatial expression patterns of an ethylene biosynthetic gene, *V. faba 1-aminocyclopropane-1-carboxylic acid* (*ACC*) *synthase 2* (*VfACS2*), demonstrated that a rapid burst in ethylene biosynthesis coincided with adaxial epidermal cells becoming competent to form wall ingrowth papillae (Zhou et al., 2010). The ethylene burst was primarily localized to adaxial epidermal cells and was driven by auxin (Zhou et al., 2010; see Figure 4). Protein abundance and gene expression profiles of key downstream ethylene signaling components, ethylene insensitive 3 (VfEIN3-1) and three ethylene response factors, showed that ethylene-regulated signaling events were epidermal cell specific and occurred rapidly before wall ingrowth induction (Zhou et al., 2010). Intracellular concentrations of glucose, sensed through a hexokinase-dependent pathway, determined progression of the ethylene-signaling cascade through EIN3 to activate gene expression leading to wall ingrowth formation (Andriunas et al., 2011; see Figure 4).

Upon ethylene signaling, *V. faba* respiratory burst oxidase homologues (*Vfrbohs*) are rapidly induced (Andriunas et al., 2012) and generate two sequential bursts in SOD-dependent extracellular H_2_O_2_ production spatially restricted to outer periclinal walls of epidermal cells (Andriunas et al., 2012; Xia et al., 2012). The extracellular H_2_O_2_ signal serves two functions in regulating ingrowth wall formation in adaxial epidermal cells. First, H_2_O_2_ activates, in a yet-to-be-determined mechanism, cell wall biosynthetic activity. Second, the H_2_O_2_ signal functions as a positional cue that directs trafficking of cell wall biosynthetic machinery to the outer periclinal wall to catalyze assembly of the polarized uniform wall, the attenuation of which impacts subsequent wall ingrowth formation (Andriunas et al., 2012; Xia et al., 2012; see Figure 4). A currently unknown mechanism re-directs deposition of cell wall material from laying down the uniform wall to one in which wall deposition is restricted to loci from which wall ingrowth papillae arise (Figure 4).

Together these findings provide insight into the possible sequence of events leading to induction of TCs in abaxial epidermal cells of *V. faba* cotyledons developing *in planta* (Offler et al., 1997; Figure 4). Here, abaxial epidermal TCs *trans*-differentiate in a progressive basipetal wave as expanding cotyledons contact and crush the innermost layers of thin-walled parenchyma cells of the seed coat (Offler et al., 1989; Harrington et al., 1997; see Figure 4). In this context, the detected sensitivity of *VfACS2* expression in seed coats to mechanical wounding (Zhou et al., 2010) suggests that crushing of their innermost cell layers *in planta* (Harrington et al., 1997) could cause a spike in ethylene production (Zhou et al., 2010; Figure 4). In turn, seed coat-derived ethylene might drive an accompanying ethylene burst in opposing abaxial epidermal cells of the cotyledons through autoregulated expression of *VfACS2* (Chang et al., 2008) that initiates their *trans*-differentiation to a TC morphology (Zhou et al., 2010). Coincident with the epidermal-cell specific ethylene burst, their intracellular glucose levels decline (Borisjuk et al., 1998) as a result of crushing seed coat cells in which an extracellular invertase is specifically expressed (Weber et al., 1996). Lowered intracellular glucose levels, sensed through a hexokinase-dependent pathway, removes glucose repression of EIN3 abundance thus allowing an ethylene-signaling cascade through EIN3 to initiate *trans*-differentiation to a TC morphology (Andriunas et al., 2011; Figure 4). Consistent with this assertion, endogenously produced ethylene, released from an experimentally-imposed mechanical wounding of seed coats and pod walls, caused normally uncommitted adaxial epidermal cells to form ingrowth walls (Zhou et al., 2010). Upon ethylene signaling, downstream targets are rapidly induced (Dibley et al., 2009), including a polarized NADPH oxidase- and superoxide dismutase (SOD)-dependent burst in extracellular H_2_O_2_ production (Andriunas et al., 2012; Xia et al., 2012), to culminate in constructing polarized ingrowth walls in the abaxial epidermal cells (Figure 4).

Based on the scenario outlined above, the role of endogenous auxin as part of the signaling complex, detected *in vitro* (Dibley et al., 2009; Zhou et al., 2010), is currently unclear for *in planta trans*-differentiation of abaxial epidermal cells of *V. faba* cotyledons to a TC morphology. However, some hints are beginning to emerge from studies of *in planta trans*-differentiation to a TC morphology of basal endosperm cells of developing maize kernels. For instance, attenuated ingrowth wall formation in the maize *mn-1* mutant, which causes loss of catalytic activity of an extracellular invertase (INCW2) localized to basal endosperm cells, has been accounted for by a lowered glucose signal, sensed through a hexose kinase-dependent pathway, driving a positive regulation of auxin biosynthesis and hence auxin levels in these cells (LeClere et al., 2010). Similar conclusions linking auxin maxima and basal endosperm cell *trans*-differentiation to a TC morphology have been obtained in another maize mutant with lowered auxin levels detected in their basal endosperm cells (*de-B18* mutant—Forestan et al., 2010). Significantly, TC formation in attached wild type maize kernels (Forestan et al., 2010) and cultured *V. faba* cotyledons (Dibley et al., 2009) share a common feature of a delocalized distribution of PINFORMED1 (PIN1) proteins in their *trans*-differentiating cells. This observation leads to the speculation that, in the absence of wound-induced ethylene formation as found for maize kernels and cultured *V. faba* cotyledons, auxin maxima in the TC founder cells function to initiate the ethylene signal. Consistent with this assertion is an up-regulated expression of genes encoding ethylene metabolism enzymes and ethylene responsive transcription factors in barley endosperm cells undergoing TC formation (Thiel et al., 2008, 2012). A respiratory burst oxidase is co-ordinately expressed in developing barley endosperm TCs (Thiel et al., 2008, 2012), implicating a regulatory role for reactive oxygen species (Figure 4 and associated text). Ethylene and ROS may also participate in regulating maize basal endosperm TC formation since *BETL* and *MEG1* share homology with defensin-like genes (Thompson et al., 2001) and their expression is known to be responsive to ethylene (Wang et al., 2002) and/or ROS (Gadjev et al., 2006). Overall this analysis suggests that the signals and signaling pathways responsible for regulating induction of TCs in developing seeds could be conserved across mono- and eudicots.

## Does an auxin-ethylene-ROS signaling cascade regulate phloem transfer cell *trans*-differentiation?

Given that TCs are ubiquitous within the plant kingdom, the potential exists for any cell type to *trans*-differentiate into a TC morphology. For phloem cells, this potential is illustrated by TC induction following invasion of the plant host vasculature by root-knot nematodes (Hoth et al., 2008), stem and root parasites (Hibberd and Jeschke, 2001), nodules of nitrogen-fixing bacteria (Pate and Gunning, 1972) and leafhoppers (Ecale-Zhou and Backus, 1999 and see Section Phloem Invaders). Induction of TC *trans*-differentiation undoubtedly relies on a specific cascade of coordinated signals that, in part, may also depend upon particular spatial relationships with surrounding cells. We explore this question using the auxin-ethylene-ROS cascade regulating TC induction in developing seeds as a framework (see Section Signals Regulating Transfer Cell *Trans*-Differentiation—Developing Seeds Show the Way and Figure 4). These putative TC inductive signals are evaluated against findings drawn from formation of phloem TCs, or cells adjacent to the phloem, in response to invasion by biotrophs, in particular, root-knot nematodes (Hoth et al., 2008) and, to a lesser extent, nitrogen-fixing bacteria (Pate and Gunning, 1972 and see Section Functional Role of Transfer Cells in Phloem Transport). Finally this information is assembled into speculative scenarios that may account for TC formation in the collection and transport phloem for each putatively identified signal.

### Auxin maxima drive de-differentiation of founder cells

The most convincing evidence for auxin (IAA) maxima functioning as a developmental signal initiating *trans*-differentiation to a TC morphology (see Section Signals Regulating Transfer Cell *Trans*-Differentiation—Developing Seeds Show the Way) within or proximal to the phloem comes from studies of nematode giant cell development. For instance, a spike in auxin concentration occurs as early as 18 h post-infection in giant cell progenitor cells which then declines from 42 h post-infection to being absent at 10 days post-infection (Karczmarek et al., 2004). The auxin spike in developing giant cells likely results from an upregulated expression of auxin efflux carriers (Hammes et al., 2005; Damiani et al., 2012) and auxin biosynthetic genes (Barcala et al., 2010) that collectively leads to a downstream auxin-signaling cascade (Barcala et al., 2010). Similar events take place in syncytia-forming nematodes where it has been shown that auxin signaling mutants significantly lower establishment of effective nematode infections (Grunewald et al., 2009). Early formation of nitrogen-fixing nodules in legumes is also linked with auxin maxima in nodule primordial and pericycle cells (Grunewald et al., 2009) with the latter cells subsequently *trans*-differentiating to a TC morphology (Pate and Gunning, 1972). In both cases, auxin maxima appear to function as a cell de-differentiation step to form a population of TC founder stem cells (Grunewald et al., 2009).

The relationship between auxin maxima and TC formation broadly can be mapped to, and account for, TC formation throughout the phloem transport system. Here auxin maxima and TC formation co-occur in developing minor vein networks of collection phloem (Gunning et al., 1968; Aloni et al., 2003). In the case of transport phloem, the relationship between auxin maxima and TC formation tends to be localized to stem nodes (Gunning et al., 1970; Aloni, 2010) and to sites of lateral root formation (Aloni, 2010). In developing leaves of *Pisum sativum*, CC/TCs differentiate concurrently with the minor vein network (Gunning et al., 1970) with development of the latter regulated by a canalized flow of auxin emanating from trichomes and mesophyll cells (Aloni et al., 2003). If the cell-specific localization of auxin maxima observed for wall ingrowth formation in nematode giant cells (see above) and developing seeds (see Section Signals Regulating Transfer Cell *Trans*-Differentiation—Developing Seeds Show the Way) are an universal requirement for TC formation, this may account for why only 20 to 30 percent of Angiosperm species form TCs in their collection phloem (see Table 1).

### Ethylene—a dispensible intercellular inductive signal?

Successful establishment of a biotrophic relationship with a plant host, be it pathogenic (nematodes) or symbiotic (nitrogen-fixing bacteria), involves suppression of host defenses coupled with a localized re-programming of host cell development (e.g., Portillo et al., 2013). Ethylene functions in both these processes as a defense (Broekaert et al., 2006) and developmental (Vanstraelen and Beková, 2012) signal. Consistent with suppression of host defenses, transcriptomic evidence points to down regulation of ethylene biosynthesis (Barcala et al., 2010), downstream components of the ethylene transduction pathway (Caillaud et al., 2008) including ethylene response factors (Portillo et al., 2013) during early phases (3 and 7 days post infection) of nematode giant cell development. In contrast, elevated rates of ethylene biosynthesis coincide with the rapid phase of gall growth (9 to 16 days post infection) that was increased further upon exposure to an exogenous ethylene source (Glazer et al., 1983). A balance between suppression of host defense responses and re-programming host cell development likely depends upon a sophisticated mechanism. A hint that such a mechanism exists has been revealed in a detailed temporal expression analysis of six ethylene response factor (ERF) isoforms of *Lotus japonica* roots during infection with a nitrogen-fixing bacterium. The findings show that *ERF* expression patterns were isoform specific and that expression of *LjERF1* had a positive effect on nodule formation (Asamizu et al., 2008). This suggests that ethylene regulation of nodule formation alters across nodule development. Relevant to development of pericycle TCs located proximal to nodule phloem strands, ethylene biosynthetic gene expression is upregulated in pericycle cells facing protophloem poles of the root stele (Heidstra et al., 1997).

It is uncertain whether ethylene functions in inducing TC development in collection and transport phloem at nodes and sites of lateral root formation. Within collection phloem, jasmonic acid selectively upregulates, but does not induce, deposition of polarized wall ingrowths in phloem parenchyma TCs (Amiard et al., 2007). This observation suggests that ethylene functions as an intercellular signal for TC development as found for developing *V. faba* cotyledons where its origin is maternal and switches on filial epidermal cells to *trans*-differentiate into TCs by activating production of an extracellular ROS signal (Andriunas et al., 2012; Xia et al., 2012). In this context, ethylene may be superfluous for the non-polarized wall ingrowths formed in nematode giant cells (Hoth et al., 2008) and CCs of collection phloem (see Table S1) if an intracellular ROS signal was operative within these cells (see next section). However, it is pertinent to note that ethylene biosynthetic machinery is expressed in developing SEs (Gallie et al., 2009) at a stage that coincides with the period during which CCs *trans*-differentiate to a TC morphology in pea minor veins (Gunning et al., 1968).

### ROS—activator of, and polarity signal for, uniform wall deposition

ROS falls into the same category as ethylene in serving dual functions of mounting defense responses (so-called oxidative burst) and re-programming development during establishment of a host plant/biotrophic relationship. Thus, a strong burst in extracellular ROS production by host cell NADPH oxidases and peroxidases accompanies root knot nematode invasion at 12 to 24 h post invasion before declining to lower levels during giant cell formation (Melillo et al., 2006, 2011; Das et al., 2008). During the latter period, peroxidases are expressed in giant cells (Severino et al., 2012) that could regulate intracellular ROS levels to induce cell wall biosynthesis linked with ingrowth wall formation (Andriunas et al., 2012; Xia et al., 2012). Intracellularly-produced ROS in nodule founder cells (Lee et al., 2005) is essential for initiating nodule primordia in developing legume-*Rhizobium* symbioses (D'Haeze et al., 2003). This intracellular ROS may diffuse to, and induce, adjacent pericycle cells to form ingrowth walls (Pate and Gunning, 1972).

**Figure 5:**
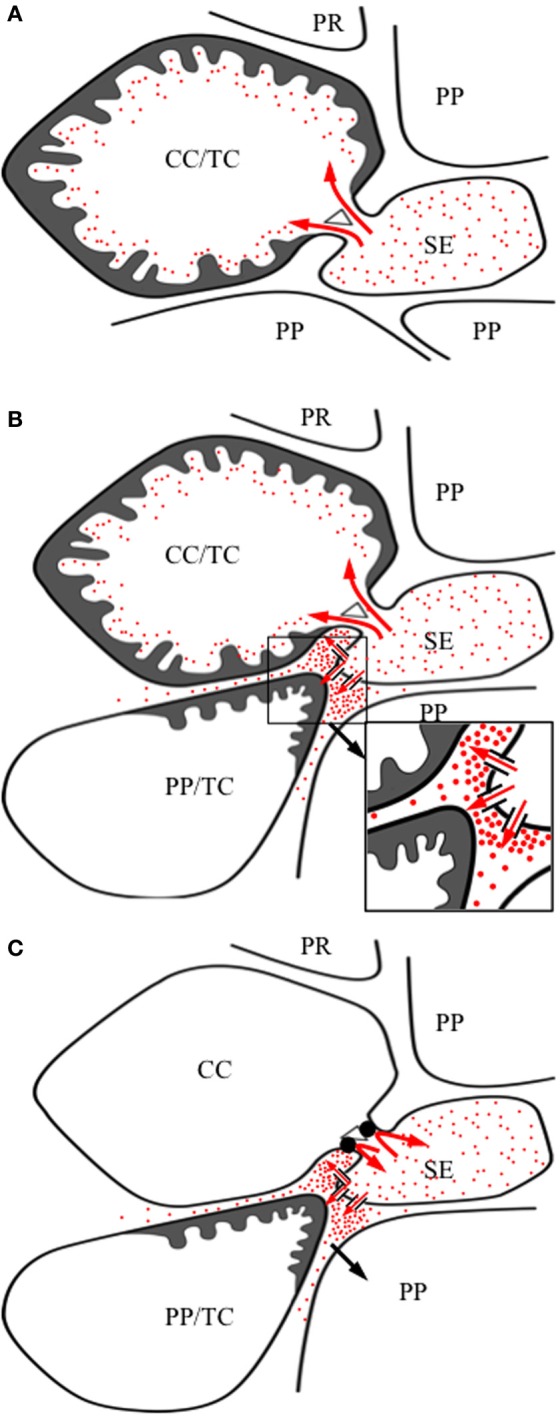
**Speculative model illustrating reactive oxygen species (ROS) mediated regulatory mechanisms that may account for observed patterns of inter- and intracellular formation of ingrowth walls (dark gray) in collection phloem companion cells (CC) and phloem parenchyma cells (PP) undergoing *trans*-differentiation to a transfer cell (TC) morphology.** In all cases, developing sieve elements (SEs), undergoing partial programmed cell death, generate an intracellular ROS signal. **(A)** CC/TC *trans*-differentiation: SE-produced intracellular ROS (red dots) diffuses (thick red arrows) into CC, through interconnecting plasmodesmata, to induce formation of a non-polarized ingrowth wall covering the entire CC wall. **(B)** CC/TC and PP/TC *trans*-differentiation: an intracellular ROS signal induces ingrowth wall formation as described in **(A)**. In addition, intracellular ROS, released from developing SEs through plasma membrane aquaporins abutting adjacent PP (thin red arrows) into the shared SE/PP cell wall space, elicits a polarized formation of ingrowth walls proximal to SEs. **(C)** PP/TC *trans*-differentiation: A transient and excessive intracellular generation of ROS by developing SEs gates plasmodesmata interconnecting CCs closed (filled in) thus preventing transmission of the ROS signal (curved red arrows) across the developmental period in which CCs are otherwise ROS-responsive. Ingrowth wall formation in PPs occurs as described in **(B)**.

Developing collection and nodal transport phloem are exposed to a number of ROS sources including bundle sheath cells exposed to high light (Galvez-Valdivieso et al., 2009) and differentiating SEs (Beers and McDowell, 2001) and tracheary elements (Karlsson et al., 2005) undergoing partial or complete programmed cell death, respectively. Bundle sheath cell produced extracellular ROS (H_2_O_2_) migrates through cell walls of adjoining vascular parenchyma cells (Galvez-Valdivieso et al., 2009). However, ROS generated by bundle sheath cells is unlikely to function as a TC-specific signal. First, ROS diffusing from bundle sheath cells does not account for ingrowth wall deposition in phloem parenchyma cells commonly polarized to SE poles (see Table S1). Second, bundle sheath cells only generate ROS in fully-expanded leaves whilst *trans*-differentiation of phloem parenchyma and CCs to a TC morphology is completed prior to full expansion (Gunning and Pate, 1974). Never-the-less, bundle sheath cell ROS could be a component of the regulatory system controlling the extent to which ingrowth walls develop (Amiard et al., 2007 and see below). ROS arising from developing tracheary elements coincides with TC *trans*-differentiation, but is unlikely to be a key player as, similar to bundle sheath cell ROS, it would not provide positional information for the polarized deposition of ingrowth walls in phloem parenchyma cells. Thus, by elimination, this analysis brings focus to differentiating SEs as the most likely source of a ROS signal inducing TC formation by CCs and phloem parenchyma cells. A high plasmodesmal connectivity between sister SEs and CCs (Patrick, 2012) offers a symplasmic delivery route for ROS to reach CCs (see above for nematode giant cells). Symplasmic delivery of ROS is consistent with the non-polarized deposition of ingrowth walls in collection phloem CCs (see Table S1 and Figure 5A). In contrast, wall ingrowth formation polarized to tips of phloem parenchyma cells abutting SEs cannot be accounted for by a SE signal delivered symplasmically. A symplasmic connection between these cell types is absent (Patrick, 2012). Even if there were a limited symplasmic connectivity, an intracellular ROS signal would not be capable of providing positional information to the phloem parenchyma cells. An extracellular ROS signal abutting phloem parenchyma cells could be released to the shared cell wall from SEs by transport through clusters of aquaporins (Bienert et al., 2007). This would generate a localized ROS signal that would collectively induce cell wall biosynthesis as well as polarized deposition of the ingrowth wall in the abutting phloem parenchyma cell (Figure 5B) functioning along lines comparable to extracellular ROS regulating polarized wall formation during tip growth of root hairs and pollen tubes (Swanson and Gilroy, 2010). If the suggested requirement for aquaporins being positioned in SE plasma membranes applies, this could account for the low proportion of Angiosperm species developing phloem parenchyma TCs in combination with CC/TCs (see Table 1). This model does not account for phloem parenchyma cells alone forming TCs. This outcome would depend on blocking symplasmic movement of intracellular ROS from SEs to CCs (Figure 5C). In this case, it is pertinent to note that elevated intracellular ROS levels caused plasmodesmata to gate closed while relatively lower ROS concentrations increased plasmodesmal permeability (Rutschow et al., 2011). Whatever the mechanism, the inductive mechanism regulating phloem parenchyma cells to *trans*-differentiate to a TC morphology is likely to be unique to these cells accounting for the unusually low number of species forming phloem parenchyma TCs alone in the collection phloem (see Table 1).

Regulation of the extent of TC formation in phloem parenchyma cells in response to abiotic stress supports a potential role for ROS in ongoing ingrowth wall development. For example, wall ingrowth deposition in leaf phloem parenchyma cells of *Senecio vulgaris* and Arabidopsis was enhanced following exposure to high light (Amiard et al., 2007) possibly driven by bundle sheath cell generated extracellular H_2_O_2_ (Galvez-Valdivieso et al., 2009). This H_2_O_2_ signal could amplify ingrowth wall biosynthesis in phloem parenchyma TCs as demonstrated by a lack of an enhanced wall ingrowth development when *Gigantea* mutants of Arabidopsis are exposed to high light (Edwards et al., 2010) possibly due to their enhanced ability to detoxify/scavenge ROS (Cao et al., 2006). Conversely, an Arabidopsis vitamin E mutant supporting depressed levels of tocopherol, a key ROS scavenger, would likely have elevated ROS levels that would account for their increased wall ingrowth formation (Maeda et al., 2006, 2008).

### The unknown signal guiding localized deposition of wall ingrowths

The requirement for a signal orchestrating deposition of wall ingrowths necessitates that the signal creates localized domains within the plasma membrane in which wall-building machinery is assembled. To our knowledge, there is no known example for such a signal in the plant kingdom. However, such an organization exists in animal cells. For example, localized Ca^2+^ signals, formed by the combined activities of plasma membrane and ER Ca^2+^ channel activity, control positioning of growth cones at tips of growing axons (Tojima, 2012). In this context, examples exist for plant cells where transporters are clustered into microdomains on their plasma membranes (see Eggert et al., 2013 this volume). Thus, clusters of plasma membrane Ca^2+^ channels linked with Ca^2+^ effluxers could generate localized Ca^2+^ signals in cells *trans*-differentiating to a TC morphology. Significantly, Ca^2+^ transporters are expressed at feeding sites of root-knot nematodes (Hammes et al., 2005). The collective activity of these transporters would sculptor localized Ca^2+^ signals (McAinsh and Pittman, 2008) to direct localized delivery of wall building machinery through re-structuring the cytoskeleton (Hepler et al., 2012) to assemble wall ingrowth papillae.

## Conclusions

In exploring the intimate association between TCs and phloem transport some interesting observations have emerged that identify profitable areas to advance understanding of drivers regulating resource allocation and thus inform intervention strategies to increase crop productivity. For instance, the adaptive significance of nodal TCs is highlighted by the percentage of Angiosperm species forming TCs at nodes far exceeding that for leaf minor veins (Tables 1 cf., 2). Nodal TCs are positioned to play significant roles in regulating resource allocation to optimize realization of reproductive potential (see Sections Key Apoplasmic Steps in the Phloem Transport Pathway, Broad Evolutionary Trends in Relation to Cellular Location of Transfer Cells within Phloem Transport Pathways, and Functional Role of Transfer Cells in Phloem Transport) and hence crop yield. However, apart from the extensive survey by Gunning et al. (1970), there is scant knowledge of the mechanisms regulating nodal TC induction and those underpinning their resource transport functions. The future challenge will be to identify and develop tractable experimental systems to address these challenging questions. Similarly for post-phloem transport, an analysis of TC occurrence at symplasmic disjunctions located at interfaces between generations in Bryophytes (gametophyte/sporophyte) and developing Angiosperm seeds (maternal/filial) highlighted a major evolutionary shift from an equal TC presence in both generations for Bryophytes to one strongly biased to a filial location in Angiosperm seeds during their storage phase of development (Table 3). Thus, a comprehensive understanding of why filial TCs have become dominant in developing seeds may open up opportunities to engineer increases in crop yield.

While there is an emerging body of evidence demonstrating a positive relationship between wall ingrowth amplification of plasma membrane surface areas and resource transport rates of phloem loading and unloading, the functional contribution of transporters targeted to these membranes remains essentially untested (see Section Functional Role of Transfer Cells in Phloem Transport). Similarly, current knowledge of inductive signals initiating *trans*-differentiation to a TC morphology is fragmentary and is best understood for developing seeds (see Sections Signals Regulating Transfer Cell *Trans*-Differentiation—Developing Seeds Show the Way and Does an Auxin-Ethylene-Ros Signaling Cascade Regulate Phloem Transfer Cell *Trans*-Differentiation?). Arabidopsis phloem parenchyma TCs are being used to address these knowledge gaps for collection phloem TCs. However, the peculiarities of ingrowth wall development in the less common phloem parenchyma TCs (see Sections Broad Evolutionary Trends in Relation to Cellular Location of Transfer Cells within Phloem Transport Pathways and Does an Auxin-Ethylene-Ros Signaling Cascade Regulate Phloem Transfer Cell *Trans*-Differentiation?) indicates caution will be needed in extrapolating conclusions to inductive mechanisms for CC/TCs. There is an even greater dearth of information about regulatory mechanisms controlling induction and intracellular targeting of membrane transporters in developing phloem TCs. Arguably the root-knot nematode system affords the best opportunity to study inductive signaling of TCs associated with the phloem (see Section Does an Auxin-Ethylene-Ros Signaling Cascade Regulate Phloem Transfer Cell *Trans*-Differentiation?). Transcriptomic analyses are identifying cohorts of TC specific genes putatively responsible for organizing and constructing their ingrowth walls. At the mechanistic level for constructing ingrowth walls (see Section Signals Regulating Transfer Cell *Trans*-Differentiation—Developing Seeds Show the Way), the crucial process to enhance plasma membrane surface area and thus membrane transport capability, is to determine the signal(s) defining loci for wall ingrowth deposition.

Since all plant species have the genomic capacity for TC *trans*-differentiation, the potential exists for development of this functionally important phloem cell type in strategic locations for resource flow in species where TCs are absent. A combination of broad transcriptomic analyses, directed molecular analysis and mechanistic cell biology studies will be needed to achieve such an outcome.

### Conflict of interest statement

The authors declare that the research was conducted in the absence of any commercial or financial relationships that could be construed as a potential conflict of interest.

## Dedication

We dedicate this review to two lads from Queens University Belfast, Brian ES Gunning and John S Pate, who four decades ago made transfer cells their passion. Their legacy in this field of transfer cell biology is clearly evident in this review. Their extensive surveys, exquisite electron micrographs and insightful predictions continue to provide the foundation for the more recent cellular and molecular studies that are attempting to unravel the mysteries of this uniquely specialized and functionally significant cell type.
