# Supplementary material for: Intersection of transfer cells with phloem biology—broad evolutionary trends, function, and induction
Source: Front Plant Sci. 2013 Jul 1;4:221. doi: 10.3389/fpls.2013.00221 (PMC3696738; doi:10.3389/fpls.2013.00221)
Supplement: Supplementary file 1 [file DataSheet1.DOCX]

**Supplemental Tables**

**Table S1** A compilation of reports of collection phloem transfer cells of Angiosperms detailing transfer cell type, ingrowth wall architecture, the spatial relationships between transfer cells and adjacent cells and extent of polarization of the ingrowth wall. Only reports that allowed this information to be gathered from published images are included in the table. BSC, bundle sheath cell; CC, companion cell; CC, companion cells; CC/TC, companion cell transfer cell; MIC, modified intermediary cell; ND, no data; PPC, phloem parenchyma cell, PP/TC, phloem parenchyma transfer cell; SE, sieve element; TC, transfer cell; TE, treachery element; VE, vessel element.

| **PHLOEM TYPE: Collection** | | | | | |
| --- | --- | --- | --- | --- | --- |
| **Plant Species** | **Transfer Cell Type** | **Wall Ingrowth Type(s)** | **Associated Cells**  **(TC adjacent to)** | **Wall Ingrowths Polarized**  **+ or -** | **References** |
| **MONOCOTYLEDON** | | | | | |
| *Allium cepa* L. | CC | Reticulate | SE, BSC and CC/TC | **-** | Lalonde *et al*., (2001). (Fig. 1b); Wilson *et al*., (1985). (Fig. 10) |
| *Triticum aestivum* | PPC | Reticulate | ND | ND | Kuo et al., (1972). |
| *Zostera nana* | PPC and BSC | Reticulate | PPC/TC, SE and CC | **+** to:  SE, PPC/TC | Pate and Gunning, (1969). (Fig. 9) |
| **EUDICOTYLEDON** | | | | | |
| *Ammobium alatum* | CC  PPC | Reticulate  ditto | SE and each other type of TC  ditto | **-**  **+** to:  SE | Gunning and Pate, (1969). (Fig. 10) |
| *Anacyclus pyrethrum* | CC  PPC | Reticulate  ditto | SE and each other type of TC  ditto | **-**  (No WIs abut SE)  **+** to:  SE and CC/TC cell wall neighbouring to SE | Pate and Gunning, (1969). (Fig. 2) |
| *Arabidopsis thaliana* | PPC | Reticulate | SE and CC | **+** to:  SE | Amiard *et al*., (2007). (Fig. 1e); Haritatos *et al*., (2000). (Fig. 3) |
| *Asarina barclaiana* | CC | Reticulate | MIC and SE | **-**  (Few abut SE) | Voitsekhovskaja *et al*., (2006). (Fig. 2B) |
| *Asarina scandens* | CC  MIC | Reticulate  ditto | SE and MIC  BSC | **-**  **+** to:  BSC | Turgeon *et al*., (1993). (Fig. 3A)  (Fig. 3B) |
| *Cymbalaria muralis* | CC | Reticulate | SE, PPC and other CC/TC | **-**  (No WIs abut SE) | Turgeon et al., (1993). (Fig. 3D) |
| *Helianthemum* | CC | Flange | XE, SE and PPC | **-** | Jones and Gunning, (1976). (Fig. 2) |
| *Impatiens balsamina* Royle | CC  PPC | Reticulate  ditto | SE and each other TC  ditto | **-**  (Few abut SE)  **+** to:  SE | Gunning and Pate, (1969). (Fig. 9) |
| *Linaria maroccana* | CC | Reticulate | SE and CC/TC | **-**  (Few abut SE) | Turgeon *et al*., (1993). (Fig. 4) |
| *Lupinus* | CC | Reticulate | SE | **-** | Gunning *et al*., (1968). (Fig. 7) |
| *Medicago sativa* cv *Gabes* | CC | Reticulate | SE and CC/TC | **-**  (No WIs abut SE) | Boughanmi *et al*., (2003). (Fig. 3A) |
| *Nemesia strumosa* | CC | Reticulate | SE and MIC | **-** | Turgeon et al., (1993). (Fig. 1C) |
| *Phodochiton atrosanguineum* | CC | Reticulate | ND | ND | Turgeon et al., (1993). |
| *Pisum arvense* | CC | Reticulate | SE, TE, BSC and other CC/TC | **-** | Gunning et al, (1968). (Fig. 1) |
| *Pisum sativum* | CC | Reticulate | SE, BSC and PPC | **-**  (Few abut SE) | Amiard et al., (2007). (Fig. 1d); |
| *Plantago major* | PPC | Reticulate | BSC, CC and SE | **+** to:  SE | Reidel et al., (2009). (Fig. 1D) |
| *Senecio vulgaris* | CC  PPC | Reticulate  Reticulate | SE and each other TC  ditto | **-**  **+** to:  SE | Amiard et al., (2007). (Fig. 1 c&f); Pate and Gunning, (1969) (Fig. 3); |
| *Sonchus oleraceus* | CC | Reticulate | SE, TE, PPC, BSC and lactifer | **-**  (Few abut SE) | Fisher, (1991). (Fig. 7) |
| *Trifolium alexandrinum* | CC | Reticulate | PPC, SE, mesophyll cell and other CC/TC | **-** | Winter, (1982). (Fig. 5a) |
| *Vicia faba* | CC | Reticulate | BSC, SE, PPC and VE | **-**  (Few abut SE) | Bourquin et al., (1990). (Fig. 5A) |
| *Zinnia elegans* | CC | Reticulate | SE and CC/TC | **-**  (Few abut SE) | Oparka and Turgeon, (1999). (Fig. 1A) |

**Table S2** A compilation of the occurrence of transfer cells associated with the transport phloem in leaves, stems and roots of Angiosperms detailing transfer cell type, ingrowth wall architecture, the spatial relationships between transfer cells and adjacent cells and extent of polarization of the ingrowth wall. Because of the close functional relationship between xylem and phloem transfer cells both are recorded. Only reports that allowed this information to be gathered from published images are included in the table. CC, companion cell; CC/TC, companion cell transfer cell; MXE, metaxylem element; ND, no data; PPC, phloem parenchyma cell, PP/TC, phloem parenchyma transfer cell; PXE, protoxylem element; SE, sieve element; TC, transfer cell; TE, treachery element; VE, vessel element; XE, xylem element; XP, xylem parenchyma cell; XPC/TC, xylem parenchyma cell transfer cell.

| **PHLOEM TYPE: Transport** | | | | | |
| --- | --- | --- | --- | --- | --- |
| **Plant Species** | **Transfer Cell Type** | **Wall Ingrowth Type(s)** | **Associated Cells**  **(TC adjacent to)** | **Wall ingrowths polarized**  **+ or -** | **References** |
| **MONOCOTYLEDON** | | | | | |
| LEAF | | | | | |
| *Phyllospadix scouleri* | PPC in leaf median vein | Reticulate | Nacreous walled SE, CC and other PP/TC | **+** to:  Nacreous walled SE | Barnabas, (1994). (Fig. 13) |
| *Tradescantia virginiana* | XP in departing leaf trace | Reticulate | XE and each other XP/TC | **+** to:  XE | Gunning *et al*., (1970). (Fig. 20) |
| STEM | | | | | |
| *Festuca rubra* | XP in vegetative node | Flange | XE and each other XP/TC | **-** | Gunning *et al*., (1970). (Fig. 22) |
| *Tradescantia virginiana* | Suspected CC in vascular plexus of the node  PPC in vascular plexus of the node | Reticulate  ditto | SE and each other type of TC  ditto | **-**  (Few abut SE)  **+** to:  SE | Gunning et al., (1970). (Fig. 21) |
| *Triticum aestivum* | Phloem TC in node  Xylem TC in node  Xylem TC in coleoptilar node | Reticulate  Flange  Flange | ND  ND  ND | ND  ND  ND | Zee, (1978); Busby and O’Brien, (1979). (Fig. 5) |
| ROOT | | | | | |
| *Zostera capensis* | PPC in root stele | Reticulate | SE, CC, XE and other PPC/TC | **+** to:  SE, CC, XE and other PP/TC | Barnabas and Arnott, (1987). (Fig. 21) |
| **EUDICOTYLEDON** | | | | | |
| LEAF | | | | | |
| *Trifolium alexandrinum* | CC in leaf major vein | Reticulate | PPC, SE and other CC/TC | **-** | Winter, (1982). (Fig. 3a) |
| *Zinnia elegans* | XP in leaf trace bundles | Reticulate | Other XP cells and PXE (Fig. 3B&C) | **+** to:  PXE | Dahiya *et al*., (2005).  (Fig. 3B & C) |
| STEM | | | | | |
| *Helianthemum* | XP in node | Reticulate | XE and adjacent TCs | **-** | Jones and Gunning, (1976). (Fig. 4) |
| *Hieracium florentinum* | XP in vascular bundle  XP in rhizome apices | Reticulate | MXE and XP/TCs  TE and XP/TCs | **+** to:  MXE  **+** to:  TE | Yeung and Peterson, (1974). (Fig. 16)  (Fig. 12) |
| *Lamium purpureum* | XP in cotyledonary nodes | Reticulate | XE | **+** to:  XE | Gunning *et al*., (1970). (Fig. 1&2) |
| *Lupinus albus* | XP in mid internodes | Reticulate | XE and XP/TCs | **+** to:  XE | Kuo *et al*., (1980). (Fig. 3) |
| *Macrotyloma uniflorum* | XP in mid internodes | Reticulate | XE and other XP/TCs | **+** to:  XE | Kuo *et al*., (1980). (Fig. 2) |
| *Phaseolus vulgaris* | XP in mid internodes | Reticulate | XE and other XP/TCs | **+** to:  Metaxylem vessel | Kuo *et al*., (1980). (Fig. 4) |
| *Psophocarpus tetragonolobus* | XP in mid internodes | Reticulate | XE and other XP/TCs | **+** to:  XE | Kuo *et al*., (1980). (Fig. 1) |
| *Zinnia elegans* | CC in stem bundle | Reticulate | SE and PPC | **-** | Dahiya *et al*., (2005). (Fig. 3H & I) |
| ROOT | | | | | |
| *Helianthus annuus* (decorative) | Root PPC  Root XP | Reticulate  ditto | SE; VE and other TCs  ditto | **+** to:  SE  **+** to:  VE | Ciamporova, (1993). (Fig. 6)  (Fig. 5) |
| *Helianthus annuus* | PPC adjacent to emergent lateral roots  XP adjacent to emergent lateral roots | Reticulate  ditto | SE  VE | ND  **-** | Ciamporova, (1993). (Fig. 4)  (Fig. 3) |
| *Helianthus tuberosus* | CC adjacent to emergent lateral roots  XP adjacent to emergent lateral roots | Reticulate  ditto | SE  VE | **-**  **+** ? to:  VE? | Ciamporova, (1993). (Fig. 2)  (Fig. 1) |
| *Hieracium florentinum* | CC in lateral root | Reticulate | SE and other CC/TCs | **-**  (Few abut SE) | Letvenuk and Peterson, (1976). (Fig. 11) |
| INVADERS | | | | | |
| *Glycine max* (soybean) | XP in stele adjacent to emergent root nodules | Reticulate | MXE and adjacent XP/TCs | **+** to:  MXE | Newcomb and Peterson, (1979). (Fig. 9a) |
| *Phaseolus vulgaris* (Kidney bean) | XP in stele adjacent to emergent root nodules | Reticulate | MXE and adjacent XP/TCs | **+** to:  MXE | Newcomb and Peterson, (1979). (Fig. 3) |
| *Pisum sativum* | CC in stele adjacent to effective pea nodules  Pericycle TC next to ineffective nodule | Reticulate  ditto | SE and PPC  Ineffective nodule and other pericycle TCs | **-**  (Few abut SE)  **-** | Newcomb and Peterson, (1979). (Fig. 12a)  (Fig. 13a) |
| *Vigna radiate* (mung bean) | XP in stele adjacent to emergent root nodules | Reticulate | MXE and other xylem TCs | **+** to:  MXE and other XP/TCs | Newcomb and Peterson, (1979). (Fig. 1a) |
| *Vicia faba* | Xylem pericycle TC in stele adjacent to emergent root nodules | Reticulate | ND | ND | Newcomb and Peterson, (1979). |

**Table S3 A** compilation of the position of transfer cells at the generation interface between gametophyte and sporophyte in species of Bryophytes and the maternal/filial interface in developing Angiosperm seeds at their storage phase of growth. Reports of the location of transfer cells during early embryo development in Angiosperms are also recorded. Abbreviations: CC, companion cell; BETC, basal endosperm transfer cell; TC, transfer cell.

| **PHLOEM TYPE: Release** | | | | | |
| --- | --- | --- | --- | --- | --- |
| **Plant Species** | **Transfer Cell Type** | **Wall Ingrowth Type(s)** | **Associated Cells**  **(TC adjacent to)** | **Wall ingrowths polarized**  **+ or -** | **References** |
| ***BRYOPHYTES*** | | | | | |
| *Dendroceros tubercularis* | Gametophyte cells in the placenta  Sporophyte cells in the placenta | Reticulate  Reticulate | Haustorial cell  Haustorial cell | **+** to:  Haustorial cell  **+** to:  Haustorial cell | Ligrone and Renzaglia, (1990). (Fig. 1) |
| *Ephemerum cohaerens* | Innermost layer of gametophyte vaginular cells towards foot of sporophyte  Outmost layer of sporophyte cells at peripheral region of the foot | Reticulate  Reticulate | Sporophyte TCs  Gametophyte TCs | **+** to:  Placental space  **+** to:  Placental space | Yip and Rushing, (1999). (Fig. 10) |
| *Isothecium myosuroides* | Innermost layer of gametophyte vaginular cells towards foot of sporophyte  Several layers of sporophyte cells at peripheral region of the foot | Reticulate  Reticulate | Sporophyte TCs  Gametophyte TCs | **+** to:  Placental space  **+** to:  Placental space | Alfayate et al., (2000). (Figs. 1 – 4) |
| *Leucodon canariensis* | Innermost layer of gametophyte vaginular cells towards foot of sporophyte  Several layers of sporophyte cells at peripheral region of the foot | Reticulate  Reticulate | Sporophyte TCs  Gametophyte TCs | **+** to:  Placental space  **+** to:  Placental space | Alfayate et al., (2000). (Figs. 5 - 8) |
| *Leptodon longisetus* | Innermost layer of gametophyte vaginular cells towards foot of sporophyte  Several layers of sporophyte cells at peripheral region of the foot | Reticulate  Reticulate | Sporophyte TCs  Gametophyte TCs | **+** to:  Placental space  **+** to:  Placental space | Alfayate et al., (2000). (Figs. 5 - 8) |
| *Lycopodium appressum* | Innermost layer of gametophyte vaginular cells towards foot  Outermost layer of sporophyte cells at peripheral region of the foot | Reticulate  Reticulate | Sporophyte TCs  Gametophyte TCs | **+** to:  Placental space  **+** to:  Placental space | Peterson and Whittier, (1991). |
| *Monoclea forsteri.* | Innermost layer of gametophyte vaginular cells towards foot of sporophyte  Several layers of sporophyte cells at peripheral region of the foot | Reticulate  Reticulate | Sporophyte TCs  Gametophyte TCs | **+** to:  Placental space  **+** to:  Placental space | Carafa et al., (2003).  (Fig. 2A) |
| *Neckera cephalonica* | Innermost layer of gametophyte vaginular cells towards foot of sporophyte  Several layers of sporophyte cells at peripheral region of the foot | Reticulate  Reticulate | Sporophyte TCs  Gametophyte TCs | **+** to:  Placental space  **+** to:  Placental space | Alfayate et al., (2000). (Figs. 9 - 12) |
| *Neckera inter-media* | Innermost layer of gametophyte vaginular cells towards foot of sporophyte  Several layers of sporophyte cells at peripheral region of the foot | Reticulate  Reticulate | Sporophyte TCs  Gametophyte TCs | **+** to:  Placental space  **+** to:  Placental space | Alfayate et al., (2000). (Figs. 13 - 16) |
| *Oedipodium griffithianum* | Innermost layer of gametophyte vaginular cells towards foot of sporophyte  Several layers of sporophyte cells at peripheral region of the foot | Reticulate  Reticulate | Sporophyte TCs  Gametophyte TCs | **+** to:  Placental space  **+** to:  Placental space | Ligrone and Duckett, (2011). (Figs. 1 and 2) |
| *Phaeoceros laevis* | Gametophyte placental cells | Reticulate | Gametophyte parenchyma, haustorial cell | **+** to:  Haustorial cell | Gambardella and Ligrone, (1987). |
| *Pojonatum aloides* | Several layers of sporophyte cells at peripheral region of the foot | Reticulate | Gametophyte vaginular cells | **-** | ND |
| Targionia hypophylla | Several layers of gametophyte vaginular cells towards foot of sporophyte  Outermost layer of sporophyte cells at peripheral region of the foot | Reticulate  Reticulate | Sporophyte TCs  Gametophyte TCs | **+** to:  Placental space  **+** to:  Placental space | Gambardella, (1987).  (Fig. 5)  (Fig. 6) |
| *Timmiella barbuloides* | Innermost layer of gametophyte vaginular cells towards foot of sporophyte  Outermost layer of sporophyte cells at peripheral region of the foot | Reticulate  Reticulate | Sporophyte TCs  Gametophyte TCs | **+** to:  Placental space  **+** to:  Placental space | Ligrone et al., (1982).  (Fig. 6) |
| *Treubia lacunosa* | Innermost layer of gametophyte vaginular cells towards foot of sporophyte  Several layers of sporophyte cells at peripheral region of the foot | Reticulate  Reticulate | Sporophyte TCs  Gametophyte TCs | **+** to:  Placental space  **+** to:  Placental space | Carafa et al., (2003).  (Fig. 2B) |
| ***ANGIOSPERM***  **MONOCOTYLEDON** | | | | | |
| PRE-STORAGE PHASE | | | | | |
| *Paphiopedilum delenatii* | Suspensor | Reticulate | Basal cells of suspersor | **-** | Lee et al., (2006). (Fig. 5) |
| STORAGE PHASE  Maternal | | | | | |
| *Echinochloa utilis* | Nucellar projection | Reticulate | Nucellus facing main vascular bundle | **-** | Zee and O’Brien, (1971). |
| *Hordeum vulgare* | Nucellar projection  Endosperm | Reticulate  Flange | Nucellus facing main vascular bundle  Starchy endosperm | **-**  ND | Cochrane and Duffus, (1980).  Weschke et al., (2000). (Figs. 4 and 5) |
| *Pappophorum subbulbosum* | Nucellar projection | Reticulate | ND | ND | Rost et al., (1984). |
| *Phyllospadix iwatensis* | Nucellar projection | Reticulate | Nucellus tissue | **-** | Kuo et al., (1990). |
| *Triticum aestivuum* | Nucellar projection  Endosperm | Reticulate  Flange | Nucellus facing endosperm  endosperm cavity | **-**  **-** | Zheng and Wang, (2011). (Figs 2, 3 and 4) |
| Filial | | | | | |
| *Coix lacryma-jobi* | Basal endosperm cells | Flange | Endosperm and other BETCs | **-** | Wada and Maeda, (1981). |
| *Echinchloa utilis* | Basal endosperm cells | Reticulate | Endosperm and other BETCs | **-** | Zee and O’Brien, (1971). |
| *Oryza sativa* | Basal endosperm cells | Reticulate | Endosperm and other BETCs | **-** | Hoshikawa, (1984). (Fig. 12) |
| *Pennisetum glaucum* | Basal endosperm cells | Reticulate | Endosperm and other BETCs | **-** | Fussell and Dwarte, (1980). |
| *Sorghum bicolor* | Two layers of endosperm cells adjacent to endosperm cavity | Flange | Endosperm and other BETCs | **-** | Wang et al., (2012). (Figs. 4 and 7) |
| *Zea mays* | One or two layers of outmost basal endosperm cells | Flange | Endosperm and other BETCs | **-** | Felker and Shannon (1980). (Fig. 1) |
| ***ANGIOSPERM***  **EUDICOTYLEDON** | | | | | |
| PRE-STORAGE PHASE | | | | | |
| *Euphorbia dulcis* | Synergid cells of embryo sac  Micropylar endosperm haustorium | Reticulate  Reticulate | Central cell of the embryo sac and ovule  Endosperm | **+** to:  Micropyle  **+** to:  Coenocytic endosperm | Gori and Sarfatti, (1970).  Gori, (1987). |
| [*Euphorbia helioscopia*](http://en.wikipedia.org/wiki/Euphorbia_helioscopia) | Synergid cells of embryo sac | Reticulate | Central cell of the embryo sac and ovule | **+** to:  Micropyle | Gori, (1977). |
| *Haemanthus katherinae* | Micropylar endosperm haustorium  Chalazal endosperm haustorium | Reticulate  Reticulate | Cellular endosperm  Cellular endosperm | ND  ND | Newcomb, (1978). |
| *Medicago sativa* | Synergid cells of embryo sac | Reticulate | Central cell of the embryo sac | ND | Sangduen et al., (1977). |
| *Rhinanthus minor* | Chalazal endosperm haustorium | Reticulate | Endosperm | **+** to:  Endosperm | Nagl, (1992). |
| *Sempervivum arachnoideum* | Basal suspensor cells | Reticulate | Micropylar haustorium and chalazal suspensor cells | **+** to:  Endosperm | [Kozieradzka-Kiszkurno](http://0-www.ncbi.nlm.nih.gov.library.newcastle.edu.au/pubmed?term=Kozieradzka-Kiszkurno%20M%5BAuthor%5D&cauthor=true&cauthor_uid=21644003) *et al*., (2012). |
| *Solanum nigrum* | Cells along the base of the chalazal region  Antipodal cup after antipodal cells were degenerated. | Reticulate  Reticulate | Endothelial cell  Embryo sac | **+** to:  Endothelial cell  **+** to:  Base of the antipodal cup | Briggs, (1995). (Figs. 1 - 5)  (Figs. 20 - 24) |
| *Vicia faba* | Central cell of Embryo sac  Basal suspensor cells | Reticulate  Reticulate | Antipodal cells  Chalazal suspensor cells and embryo | **-**  **+** to:  Endosperm | Jonasson and Walles, (1993). (Fig. 16)  (Fig. 19) |
| STORAGE PHASE | | | | | |
| *Gossypium spp* | Innermost seed coat cell | Reticulate grown upon flange | Seed coat, embryo | **-** | Pugh *et al*., (2010). (Fig. 1) |
| *Pisum sativum* | Innermost seed coat parenchyma cells  Abaxial epidermal cells of cotyledons | Reticulate  Reticulate | Seed coat parenchyma cells, abaxial epidermal cells of cotyledons  Seed coat parenchyma cells, cotyledon storage parenchyma cells | **+** to:  Cotyledons  **+** to:  Seed coats | Tegeder *et al*., (1999). (Fig. 1c)  (Fig. 1d) |
| *Rhizophora mangle* | Outermost layer of endosperm cells | Reticulate | Vacuolated endosperm, integument | **-** | Wise and Juncosa, (1989). (Fig. 2) |
| *Ricinus communis* | Outermost layer of endosperm cells | Reticulate | nucellus | **+** to:  Crushed cell walls from the nucellus | Greenwood *et al*., (2005). (Fig. 7) |
| *Vicia faba* | Innermost layer of seed coat parenchyma cells  Abaxial epidermal cells of cotyledons | Reticulate  Reticulate | Seed coat parenchyma cells, abaxial epidermal cells of cotyledons  Seed coat parenchyma cells, cotyledon storage parenchyma cells | **+** to:  Cotyledons  **+** to:  Seed coats | Offler *et al*., (1989)  Farley *et al*., (2000).  (Fig. 2) |
| *Vicia narbonensis* | Abaxial epidermal cells of cotyledons | Reticulate | Seed coat parenchyma cells, cotyledon storage parenchyma cells | **+** to:  Seed coats | Andriunas *et al*., (2011). (Fig. 1) |
