# Supplementary material for: Intersection of transfer cells with phloem biology—broad evolutionary trends, function, and induction
Source: Front Plant Sci. 2013 Jul 1;4:221. doi: 10.3389/fpls.2013.00221 (PMC3696738; doi:10.3389/fpls.2013.00221)
Supplement: Supplementary file 2 [file DataSheet2.DOCX]

**References for Supplemental table**

Alfayate, C. B., Estébanez, B., and Ron, E. (2000). The sporophyte-gametophyte junction in five species of pleurocarpous mosses. *Bryol.* 103, 467-474.

Amiard, V., Demmig-Adams, B., Mueh, K. E., Turgeon, R., Combs, A. F., and Adams III, W. W. (2007). Role of light and jasmonic acid signalling in regulating foliar phloem cell wall ingrowth development. *New Phytol*. 173, 722-731.

Andriunas, F. A., Zhang, H. M., Weber, H., McCurdy, D. W., Offler, C. E., and Patrick, J. W. (2011). Glucose and ethylene signalling pathways converge to regulate trans-differentiation of epidermal transfer cells in *Vicia narbonensis* cotyledons. *Plant J.* 68, 987-998.

Barnabas, A. D., and Arnott, H. J. (1987). *Zostera capensis* setchell: root structure in relation to function. *Aq. Bot.* 27, 309-322.

Barnabas, A. D. (1994). Anatomical, histochemical and ultrastructural features of the seagrass *Phyllospadix scouleri* Hook. *Aq. Bot.* 49, 167-182.

Boughanmi, N., Michonneau, P., Verdus, M-C., Piton, F., Ferjani, E., Bizid, E., and Fleurat-Lessard, P. (2003). Structural changes induced by NaCl in companion and transfer cells of *Medicago sativa* blades. *Protoplasma* 220, 179-187.

Bourquin, S., Bonnemain, J-L., and Delrot, S. (1990). Inhibition of loading of ^14^C assimilates by *p*-chloromercuribenzenesulfonic acid. Localization of the apoplastic pathway in *Vicia faba*. *Plant Physiol.* 92, 97-102.

Briggs, C. L. (1995). The initiation, development and removal of embryo sac wall ingrowths in the developing seeds of *Solanum nigrum* L. An ultrastructural study. *Ann. Bot.* 76, 429-39.

Busby, C. H., and O’Brien, T. P. (1979). Aspects of vascular anatomy and differentiation of vascular tissues and transfer cells in vegetative nodes of wheat. *Aust. J. Bot.* 27, 703-711.

Carafa, A., Duckett, J. G., Ligrone, R. (2003). The placenta in *Monoclea forsteri* Hook. and *Treubia lacunosa* (Col.) Prosk: Insights into placental evolution in liverworts. *Ann. Bot.*92, 299-307.

Ciaporova, M. (1993). Transfer cells in the vascular parenchyma of roots. *Biol Plant.* 35, 262-266.

Cochrane, M.P., and Duffus, C.M. (1980). The nucellar projection and modified aleurone in the crease region of developing caryopses of barley (*Hordeum vulgare L. var. disitichum*). *Protoplasma* 103, 361-75.

Dahiya, P., Milioni, D., Wells, B., Stacey, N., Roberts, K., and McCann, M. C. (2005). A RING domain gene is expressed in different cell types of leaf trace, stem, and juvenile bundles in the stem vascular system of *Zinnia*. *Plant Physiol.* 138, 1383-1395.

Davis, A. R., Peterson, R. L., and Shuel, R. W. (1988). Vasculature and ultrastructure of the floral and stipular nectaries of *Vicia faba* (Leguminosae). *Can. J. Bot.* 66, 1435-1448.

Farley, S. J., Patrick, J. W., and Offler, C. E. (2000). Functional transfer cells differentiate in cultured cotyledons of *Vicia faba L*. seeds. *Protoplasma* 214, 102-117.

Felker, F. C., and Shannon, J. C. (1980). Movement of C^14^-labeled assimilates into kernels of *Zea mays* L.: An anatomical examination and microautoradiographic study of assimilate transfer. *Plant Physiol*. 65, 864-70.

Fisher, D. G. (1991). Plasmodesmatal frequency and other structural aspects of assimilate collection and phloem loading in leaves of *Sonchus oleraceus* (Asteraceae), a species with minor vein transfer cells. *Am. J. Bot.* 78, 1549-1559.

Fussell, L. K., and Dwarte, D. M. (1980). Structural changes of the grain associated with black region formation in *Pennisetum americanum*. *J. Exp. Bot.* 31, 645-654.

Gambardella, R. (1987). Ultrastructure and development of the gametophyte vaginula-sporophyte foot complex in the liverwort *Targionia hypophylla* L. *Planta* 172, 431–438.

Gambardella, R., and Ligrone, R. (1987). The development of the placenta in the anthocerote *Phaeoceros laevis L*. *Planta* 172, 439–447.

Gori, P., and Sarfatti, G. (1970). Structures "transfer" du sac embryonnaire chez *Euphorbia dlucis* L. et *Euphorbia helioscopia* L. *Septieme congres International De Microscopie Electronique, Grenoble*. 447-448.

Gori, P. (1977). Wall ingrowths in the embryo sac of *Euphorbia helioscopia*. *Israel J. Bot.* 26, 202-208.

Gori, P. (1987). The fine structure of the developing *Euphorbia dulcis* endosperm. *Ann. Bot.* 60, 563-569.

Greenwood, J. S., Helm, M., and Gietl, C. (2005). Ricinosomes and endosperm transfer cell structure in programmed cell death of the nucellus during Ricinus seed development. *Proc Natl Acad Sci U S A.* 102, 2238-2248.

Gunning, B. E. S., Pate, J. S., and Briarty, L. G. (1968). Specialized “transfer cells” in minor veins of leaves and their possible significance in phloem translocation. *J. Cell Biol.* 37, C7 – C12.

Gunning, B. E. S., and Pate, J. S. (1969). “Transfer cells” Plant cells with wall ingrowths, specialized in relation to short distance transport of solutes – Their occurrence, structure, and development. *Protoplasma* 68, 107-133.

Gunning, B. E. S., Pate, J. S., and Green, L. W. (1970). Transfer cells in the vascular system of stems: taxonomy, association with nodes, and structure. *Protoplasma* 71, 147-171.

Haritatos, E., Medville, R., and Turgeon, R. (2000). Minor vein structure and sugar transport in *Arabidopsis thaliana. Planta.* 211, 105-111.

Hoshikawa, K. (1984). Development of endosperm tissue with special reference to the translocation of reserve substances in cereals III. Translocation pathways in rice endosperm. *Jap. J. Crop Sci.* 53, 153-62.

Johansson, M., and Walles, B. (1993). Functional anatomy of the ovule in broad bean, *Vicia faba* L. II. Ultrastructural development up to early embryogenesis. *Int. J. Plant Sci.* 154, 535-49.

Jones, M. G. K., and Gunning, B. E. S. (1976). Transfer cells and nematode induced giant cells in *Helianthemum*. *Protoplasma* 87, 273-279.

Kozieradzka-Kiszkurno, M., Płachno, B. J., and Bohdanowicz, J. (2012). New data about the suspensor of succulent angiosperms: Ultrastructure and cytochemical study of the embryo-suspensor of *Sempervivum arachnoideum* L. and *Jovibarba sobolifera* (Sims) Opiz. *Protoplasma* 249, 613-624.

Kuo, J., O’Brien, T. P., and Zee, S. Y. (1972). The transverse veins of the wheat leaf. *Aust. J. Biol. Sci.* 25, 721-737.

Kuo, J., Pate, J. S., Rainbird, R. M., and Atkins, C. A. (1980). Internodes of grain legumes – new location for xylem parenchyma transfer cells. *Protoplasma*. 104, 181-185.

Kuo, J., Iizumi, H., Nilsen, B. E., and Aioi, K. (1990). Fruit anatomy, seed germination and seedling development in the Japanese seagrass *Phyllospadix* (Zosteraceae). *Aq. Bot.* 37, 229-245.

Lalonde, S., Franceschi, V. R., and Frommer, W. B. (2001). [Companion Cells](http://0-onlinelibrary.wiley.com.library.newcastle.edu.au/doi/10.1038/npg.els.0002087/full). *Encyclopedia of Life Sciences*. John Wiley & Sons, Ltd.

Lee, Y. I., Yeung, E. C., Lee, N., and Chung, M. C. (2006). Embryo development in the lady's slipper orchid, *Paphiopedilum delenatii*, with emphasis on the ultrastructure of the suspensor. *Ann. Bot.* 98, 1131-1139.

Letvenuk, L. J., and Peterson, R. L. (1976). Occurrence of transfer cells in vascular parenchyma of *Hieracium florentinum* roots. *Can. J. Bot.* 54, 1458-1471.

Ligrone, R.. and Duckett, J.G. (2011). Morphology versus molecules in moss phylogeny: new insights (or controversies) from placental and vascular anatomy in *Oedipodium griffithianum*. *Plant Syst. Evol.* 296, 275-282.

Ligrone, R., Gambardella, R., and de Lucia Sposito, M.L. (1982). Ultrastructure and development of the sporophyte foot–gametophyte vaginula complex in *Timmiella barbuloides* (Brid.) Moenk. *Planta* 154, 414-425.

Ligrone, R., and Renzagnia, K. S. (1990). The sporophyte-gametophyte junction in the hornwort, *Dendroceros tubercularis* Hatt. (Anthocerotophyta). *New Phytol.* 114, 497-505.

Nagl, W. (1992). The polytenic endosperm haustorium of *Rhinanthus minor* (Scrophulariaceae): functional ultrastructure. *Can. J. Bot.* 70, 1997-2004.

Newcomb, W. (1978). The development of cells in the coenocytic endosperm of the African blood lily *Haemanthus katherinae*. *Can. J. Bot.* 56, 483-501.

Newcomb, W. and Peterson, R. L. (1979). The occurrence and ontogeny of transfer cells associated with lateral roots and root nodules in *Leguminosae*. *Can. J. Bot.* 57, 2538-2602.

Offler, C. E., Nerlich, S. M., and Patrick, J. W. (1989). Pathway of photosynthate transfer in the developing seed of *Vicia faba* L. - transfer in relation to seed anatomy. *J. Exp. Bot.* 40, 769-780.

Oparka, K. J., and Turgeon, R. (1999). Sieve elements and companion cells - traffic control centres of the phloem. *Plant Cell* 11, 739-750.

Pate, J. S., and Gunning, B. E. S. (1969). Vascular transfer cells in angiosperm leaves. A taxonomic and morphological survey. *Protoplasma* 68, 135-156.

Peterson, R. L., and Whittier, D. P. (1991). Transfer cells in the sporophyte–gametophyte junction of *Lycopodium appressum*. *Can. J. Bot.* 69, 222-226.

Pugh, D. A., Offler, C. E., Talbot, M. J., and Ruan, Y. L. (2010). Evidence for the role of transfer cells in the evolutionary increase in seed and fiber biomass yield in cotton. *Mol Plant* 3, 1075-1086.

Razem, F. A., and Davis, A. R. (1999). Anatomical and ultrastructural changes of the floral nectary of *Pisum sativum* L. during flower development. *Protoplasma* 206, 57- 72.

Reidel, E. J., Emilie, A. R., Amiard, V. A., Cheng, L. C., and Turgeon, R. (2009). Phloem loading strategies in three plant species that transport sugar alcohols. *Plant Physiol.* 149, 1601-1608.

Rost, T.L., Izaguirre de Artucio, P., and Risely, E.B. (1984). Transfer cells in the placental pad and caryopsis coat of *Pappophorum subbulosum* Arech. (Poaceae). *Am. J. Bot.* 71, 948-957.

Sangduen, N., Kreitner, G. L., and Sorensen, E. L. (1983). Light and electron microscopy of embryo development in perennial and annual *Medicago* species. *Can. J. Bot.* 61, 837-49.

Tegeder, M., Wang, X-D., Frommer, W. B., Offler, C. E., and Patrick, J.W. (1999). Sucrose transport into developing seeds of *Pisum sativum* L. *Plant J.* 18, 151-161.

Turgeon, R., Beebe, D. U., and Gowan, E. (1993). The intermediary cell: Minor-vein anatomy and raffinose oligosaccharide synthesis in the Scrophulariaceae. *Planta* 191, 446-456.

Voitsekhovskaja, O. V., Koroleva, O. A., Batashev, D. R., Knop, C., Deri Tomos, A., Gamalei, Y. V., Heldt, H-W., and Lohaus, G. (2006). Phloem loading in two Scrophulariaceae species. What can drive symplastic flow via plasmodesmata? *Plant Physiol.* 140, 383-395.

Wada, T., and Maeda, E. (1981). A comparative morphology of dorsal vascular bundles, nucellar projections and transfer cells in Gramineous caryopses. *Jap. J. Crop Sci.* 50, 199-209.

Wang, H. H., Wang, Z., Wang, F., Gu, Y. J., and Liu, Z. (2012). Development of basal endosperm transfer cells in *Sorghum bicolor* (L.) Moench and its relationship with caryopsis growth. *Protoplasma* 249, 309-321.

Weschke, W., Panitz, R., Sauer, N., Wang, Q., Neubohn, B., Weber, H., and Wobus, U. (2000). Sucrose transport into barley seeds: molecular characterization of two transporters and implications for seed development and starch accumulation. *Plant J.* 21, 455-467.

Wilson, C., Oross, J. W., and Lucas, W. J. (1985). Sugar uptake into *Allium cepa* leaf tissue: an integrated approach. *Planta* 164, 227-240.

Winter, E. (1982). Salt tolerance of *Trifolium alexandrinum L*. III. Effects of salt on ultrastructure of phloem and xylem transfer cells in petioles and leaves. *Aust. J. Plant Physiol.* 9, 239-250.

Wise, R. R., and Juncosa, A. M. (1989). Ultrastructure of the transfer tissues during viviparous seedling development in *Rhizophora mangle* (Rhizophoraceae). *Am. J. Bot.* 76, 1286-1298.

Yeung, E. C., and Peterson, R. L. (1974). Ontogeny of xylem transfer cells in *Hieracium floribundum*. *Protoplasma* 80, 155-174.

Yip, K. L., and Rushing, A. E. (1999). An ultrastructural and developmental study of the sporophyte-gametophyte junction in *Ephemerum cohaerens*. *Bryologist* 102, 179-195.

Zee, S. Y. (1978). Isolation and staining of wheat transfer cells*. Can J. Bot.* 56, 1992-1995.

Zee, S.Y., and O'Brien, T.P. (1971). Aleurone transfer cells and other structural features of the spikelet of millet. *Aust. J. Biol. Sci.* 24, 391-395.

Zheng, Y., and Wang, Z. (2011). Contrast observation and investigation of wheat endosperm transfer cells and nucellar projection transfer cells. *Plant Cell Rep.* 30, 1281-1288.
